# Investigation of Hydrazine Electrooxidation Performance of Dihydrobenzothienopyranone Derivatives

**DOI:** 10.1021/acsomega.5c12506

**Published:** 2026-03-11

**Authors:** Omruye Ozok Arıcı, Raffaella Mancuso, Bartolo Gabriele, Hilal Kivrak, Arif Kivrak

**Affiliations:** † Department of Biology, Faculty of Sciences and Arts, Eskisehir Osmangazi University, Eskisehir 26040, Turkey; ‡ Laboratory of Industrial and Synthetic Organic Chemistry (LISOC), Department of Chemistry and Chemical Technologies, 18950 University of Calabria, via Pietro Bucci 12/C, Arcavacata di Rende, Cosenza 87036, Italy; § Department of Chemical Engineering, Faculty of Engineering and Architectural Sciences, 53004 Eskisehir Osmangazi University, Eskisehir 26040, Turkey; ∥ Department of Chemistry, Faculty of Sciences, 53004 Eskisehir Osmangazi University, Eskişehir 26040, Turkey

## Abstract

This work presents the electrochemical activity of five dihydrobenzothienopyranone derivatives, **A**_**1**_–**A**_**5**_, toward the electrooxidation of hydrazine (N_2_H_4_) in alkaline media (1 M KOH + 0.5 M N_2_H_4_). All of the prepared catalysts were characterized by cyclic voltammetry (CV), chronoamperometry (CA), electrochemical impedance spectroscopy (EIS), and linear sweep voltammetry (LSV). Dihydrobenzothienopyranone (**A**_**5**_) exhibits the highest electrochemical activity, with a current density of 38.29 mA/cm^2^. It also exhibited very good long-term stability, showing constant catalytic activities, current density, and low charge transfer resistance during extended periods, with relevant features for efficient energy conversion. These findings thus constitute evidence that **A**_**5**_ might be an active but economically feasible alternative to the conventionally used transition metal-based catalysts such as Pd, Au, and Pt at the anodes of direct hydrazine fuel cells. These properties combine high stability and low cost to further make the catalyst an encouraging candidate for fuel cell technologies that are benign to the environment. These results highlight the potential of organic-based materials in fuel cell applications for the future and offer a sustainable alternative to the current metal-based systems.

## Graphical abstract

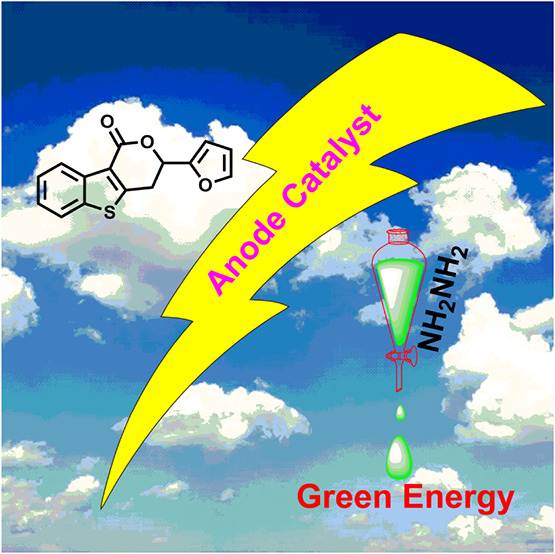

## 1 Introduction

Energy has long been one of the most essential needs of human society. The rapid growth of the global population, combined with ongoing economic development, has led to a significant rise in energy demand and has driven the search for more sustainable and efficient energy solutions. While conventional energy sources—particularly fossil fuels—have traditionally powered industry and transportation, their finite availability and harmful environmental consequences, including air pollution and global warming, have raised serious concerns about their long-term use.^1^ As a result, there has been growing interest in identifying and adopting alternative energy sources that are both environmentally friendly and sustainable. To address environmental pollution and develop eco-friendly energy conversion systems, researchers are increasingly focusing on novel energy technologies.^2^

Unlike conventional energy production methods, fuel cells offer high efficiency and generate little to no harmful emissions, making them a key component of emerging clean energy technologies.^3–5^ Various alternative fuels—such as hydrazine,^6^ glucose,^7^ methanol,^8^ ethanol,^9^ and formic acid^10^—are widely studied for their potential applications in fuel cell systems. The suitability of these fuels largely depends on the specific type of fuel cell and its intended application, with each fuel offering distinct benefits and limitations. Hydrazine’s high energy density, carbon-free oxidation profile, and low oxidation potential make it more advantageous than other liquid fuels.

Hydrazine (N_2_H_4_) is an inorganic molecule that has long been utilized as a reducing agent in chemical reactions and as a propellant in rocket propulsion systems. More recently, it has garnered significant attention as a promising fuel for low-temperature fuel cells due to its potential to generate energy through its decomposition into nitrogen and hydrogen gases. Among various alternative fuels, N_2_H_4_ stands out because of several key advantages, including its relatively low cost, ease of storage, and high energy density.

When carbon-based fuels such as methanol, ethanol, and formic acid are used in direct liquid fuel cells (DLFCs), the generation of byproducts such as carbon monoxide (CO) and carbon dioxide (CO_2_) is common. While CO_2_ emissions contribute to global warming, CO poses a more immediate threat to the operation of fuel cells by poisoning catalysts like platinum (Pt) and palladium (Pd), thereby reducing catalytic activity and leading to voltage losses in the system.^11^

In contrast, N_2_H_4_ fuel cells operate based on clean electrochemical reactions at both the anode and cathode. At the anode, N_2_H_4_ undergoes electrochemical oxidation, releasing electrons and producing nitrogen gas (N_2_) and water. These electrons flow through an external circuit, generating electrical power. Simultaneously, at the cathode, oxygen is reduced, typically in the presence of water and an alkaline electrolyte, to form hydroxide ions (OH^–^). These hydroxide ions then migrate through the electrolyte to complete the circuit, recombining with the electrons returning from the external circuit.^12^

Therefore, N_2_H_4_ fuel cells offer an efficient method of converting the chemical energy stored in N_2_H_4_ directly into electrical energy. This clean and effective energy conversion process highlights their potential in sustainable power generation applications, where balanced anode and cathode reactions work synergistically to maintain system performance.^13,14^ In essence, the fundamental operation of N_2_H_4_ fuel cells relies on the electrochemical oxidation of N_2_H_4_ at the anode and the reduction of oxygen at the cathode.

The general reaction is as follows:

According to this:

**Anodic reaction:**

1 $$N_{2}H_{4}+4OH^{-}{,,N}_{2}+4H_{2}O+4e^{-}$$

**Cathode reaction:**

2 $$O_{2}+2H_{2}O+4e^{-},,4OH^{-}$$

**Overall reaction:**

3 $$N_{2}H_{4}+O_{2}{,,N}_{2}+2H_{2}O$$

In this reaction, N_2_H_4_ undergoes dissociation at the anode, resulting in the release of hydrogen ions (protons), electrons, and nitrogen gas. This electrochemical process is key to N_2_H_4_ electrooxidation, which is being actively explored as a promising reaction for clean energy production in fuel cells. Numerous studies have been conducted recently to enhance the activity of anode catalysts for hydrazine electrooxidation, with a variety of catalysts showing improvements in both activity and stability. Notable catalysts include platinum-based catalysts such as PtCofiber/Cu,^15^ nickel-based catalysts like NiCoP/C^16^ and Ni_3_S_2_@Ni foam,^17^ silver and nickel composites such as AgNi/MWCNT,^18^ as well as copper-based catalysts such as nano-CuO/MGCE^19^ and NPCF/Cu.^20^ Other research has highlighted the performance of Au,^21^ NiMo/C,^22^ Ni–Pt/C,^23^ and Ni_3_N/Ni/NF^23^ composites, which have demonstrated promising electrooxidation activity for N_2_H_4_.^24^ Additionally, Wang et al. have established the exceptional stability and electrocatalytic efficacy of P–Cu_2_Ni/C catalysts, which are produced via phosphating.^25^ In the same way, Wen et al. found that the Ni_2_P@Ni_10_Mo/Ni–Mo–O/NF catalyst showed great activity and outstanding stability in the N_2_H_4_ electrooxidation reaction.^26^ Table 1 presents a clear comparison of the performance of these metal-based catalysts for hydrazine electrooxidation by summarizing their peak values and manufacturing techniques. Although metal-based catalysts generally show high activity, alternatives are being sought due to their high costs and limited resources. Metal–organic hybrid structures offer effective and economical solutions by combining the electronic properties of organic compounds with the catalytic properties of metals. Organic compounds have also attracted considerable interest for use in metal-based catalysts such as solar cells, electrochromic devices, and other renewable energy applications. Organic molecules are attractive to materials chemists because of their exceptional electrochemical stability, low molecular weight, flexibility, and high charge transfer capacity. These qualities make them excellent for fuel cell applications and other energy production technologies.^27–29^ The potential of organic materials to improve hydrazine electrooxidation procedures and other clean energy technologies is expanding as research into them progresses. Recently, N_2_H_4_ was electrooxidized utilizing an indole-based organic catalyst, which was shown to have the lowest charge transfer resistance and a current of 2.60 mA/cm−2 ^30^ It is emphasized that this organic compound can be used as a lower cost and environmentally friendly option compared to metal-based catalysts.

**Table 1:** Metal-Based Catalysts and Organic Catalyst for Hydrazine Electrooxidation

Catalyst	Preparation	Maximum Peak	Reference
PtCofiber/Cu	deposition	219.8 mW cm^–2^	^15^
NiFe_2_O_4_-rGO	hydrothermal method	18.9 mA/cm^–2^	^16^
Wash-CNCu	doped	1.94 mA/cm^–2^	^17^
AuPd NCs	synthesis	2.88 mA/cm^–2^	^31^
Ag/Ti	-	7.7 mA/cm^–2^	^32^
Thymol	organic synthesis	3.66 mA/cm^–2^	^33^
3-(furan-2-yl)-3,4-dihydro-1*H*-benzo[4,5]- thieno[3,2-*c*]pyran-1-one (**A**_**5**_)	organic synthesis	**38.29** mA/cm ^ **–2** ^	**In this study**

By using organic molecules as anode catalysts in fuel cells, we designed and synthesized new benzothiophene-based hybrid compounds in this study. However, the role of organic electrocatalysts in hydrazine oxidation and, in particular, the catalytic behavior of dihydrobenzothienopyrano derivatives has been largely unexplored in the literature. Dihydrobenzothienopyranone is an important compound for hydrazine electrooxidation due to its electron-transfer-facilitating structure and its catalytic effect on modified electrodes. It is also important for both energy conversion technologies and chemical sensing systems. This study aims to fill this important gap by systematically investigating the relationship between the electronic properties of this molecular structure and its performance in hydrazine electrooxidation. This work can contribute to the development of next-generation catalyst systems for future fuel cell applications. For the purpose of directly generating electricity in hydrazine fuel cells, five 3,4-dihydro-1H-benzo­[4,5]­thieno­[3,2-*c*]­pyran-1-one derivatives, **A**_**1**_–**A**_**5**_, recently reported by our research group, can be used as substitutional anode catalysts, as shown in Scheme 1.

**Scheme 1:**
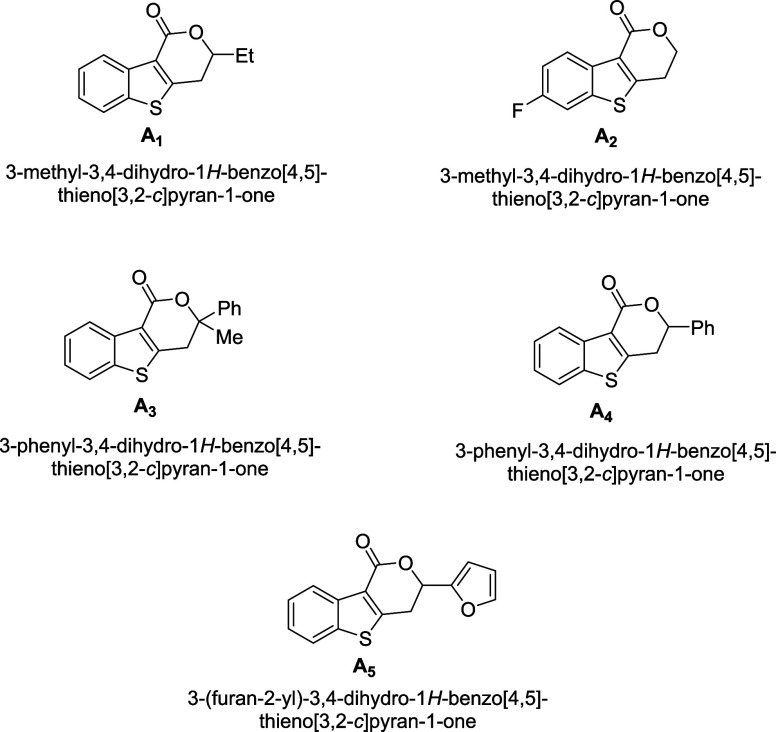
3,4-Dihydro-1*H*-benzo­[4,5]­thieno­[3,2-*c*]­pyran-1-one Derivatives **A_1_**–**A_5_** Investigated as Possible Electrocatalysts for Direct Hydrazine Fuel Cell (DHFC) Applications

## 2 Materials and Methods

### 2.1 Synthesis

Dihydrobenzothienopyranone derivatives, **A**_**1**_–**A**_**5**_, were prepared by a PdI_2_/KI-catalyzed oxidative carbonylative double cyclization of 3-(2-(methylthio)­phenyl)­prop-2-yn-1-ols, according to the published procedure.^34^ The characterization and synthesis procedures of the molecules are provided in the Supporting Information (pages S3–S29).

### 2.2 Electrochemical Studies

The organic-based catalysts **A**_**1**_–**A**_**5**_ were electrochemically tested for their activity by CV and EIS in a solution of 1 M KOH and 0.5 M N_2_H_4_ for hydrazine electrooxidation. All electrochemical analyses were carried out using a three-electrode configuration system comprising a working electrode, a reference electrode, and a counter electrode on a Gamry Interface 1010T. In all measurements, Ag/AgCl was used as the reference electrode, and a platinum wire was used as the counter electrode. A glassy carbon electrode with a diameter of 3 mm was used as the working electrode, where organic-based catalysts were deposited. First, the catalyst ink was prepared by dissolving 5 mg of the dihydrobenzothienopyranone catalyst **A** in 1 mL of the Nafion solution; then, 5 μL of the as-obtained ink was drop-cast onto the surface of the glassy carbon electrode. The electrode was then dried at room temperature in open air to evaporate the solvent. The electrocatalytic activity of hydrazine was measured within a potential window from −0.8 to 0.6 V at a scan rate of 50 mV/s. Besides, EIS was measured at an open circuit frequency of 316 kHz to calculate the electrochemical resistance of the catalysts. Thereby, this method allowed for in-depth investigation of the electrochemical behavior and catalytic activity of the organic-based catalysts in hydrazine electrooxidation reactions.

## 3 Results and Discussion

### 3.1 Electrochemical Evaluation

Using the CV approach, the dihydrobenzothienopyranone **A**_**1**_–**A**_**5**_ catalysts’ N_2_H_4_ electrooxidation activities were examined. The results, shown in Figure 1a-c, compare the CV measurements conducted in both a 1 M KOH solution and a 1 M KOH + 0.5 M N_2_H_4_ solution. As the catalysts were organic-based and lacked precious metals such as Pd, Pt, or Au, no distinct oxidation peaks were observed in the voltammograms. Consequently, the total current response was used to evaluate the catalytic performance. As illustrated in Figure 1c and summarized in Table 2, the **A**_**5**_ hybrid catalyst exhibited the highest activity among all of the tested catalysts. The specific activity, derived from the total current, was calculated to be 38.29 mA/cm^2^, demonstrating the superior electrooxidation performance of the dihydrobenzothienopyranone catalyst **A**_**5**_ in N_2_H_4_ electrooxidation. A comparison of derivatives **A_1_**–**A_5_** reveals that catalytic performance is directly related to the molecular structure. Derivative **A_5_**, which contains a furan ring and a carbonyl group, has a more extensive π-conjugation and a higher electron density distribution. This allows the hydrazine molecule to adsorb more effectively on the catalyst surface and stabilizes intermediate species. In contrast, the π-system is more restricted, and the heteroatom distribution is less favorable in derivatives **A_1_**–**A_4_**; therefore, the current density and onset potential are lower in these derivatives compared to **A_5_**. This analysis clearly demonstrates that molecular structure is a critical factor in determining electrocatalytic performance. In Figure 1d, it was observed that the currents of the **A_5_** catalyst were close to each other when compared with those of 5%Pd-**A_5_**. Figure 1e shows consecutive CVs (50 cycles) on the **A_5_** catalyst in a 1 M KOH + 0.5 M N_2_H_4_ solution. The specific activity of the **A_5_** catalyst decreased as the number of CV cycles increased. This decrease in its activity can be attributed to the accumulation of the intermediate compound on the electrode surface.^35^ The N_2_H_4_ oxidation activity on the **A_5_** catalyst decreased by approximately 52% from the first cycle to the 50th cycle. As a similar result in the literature, Ulaş reported that the N_2_H_4_ electrooxidation activities of the Pd/MWCNT/GCE catalyst decreased with consecutive CVs.^36^ Figure 1f shows the hydrazine electrooxidation behavior of the **A_5_** catalyst in a solution containing LSV 1 M KOH + 0.5 M N_2_H_4_. The total current was measured as 33.40 mA/cm^2^.

**Table 2:** Electrochemical Results of N_2_H_4_ Electrooxidation of Dihydrobenzothienopyranone Catalyst **A**_1_–**A**_5_

	Total current (mA cm^–2^)			
Catalyst	KOH	N_2_H_4_	Normal	Onset potential (V)
**A**_**1**_	0.28	21.82	21.54	0.22
**A**_**2**_	0.07	9.28	9.21	0.09
**A**_**3**_	0.006	32.27	32.26	0.25
**A**_**4**_	0.008	33.23	33.22	0.21
**A**_**5**_	0.25	**38.29**	38.04	0.20

**Figure 1:**
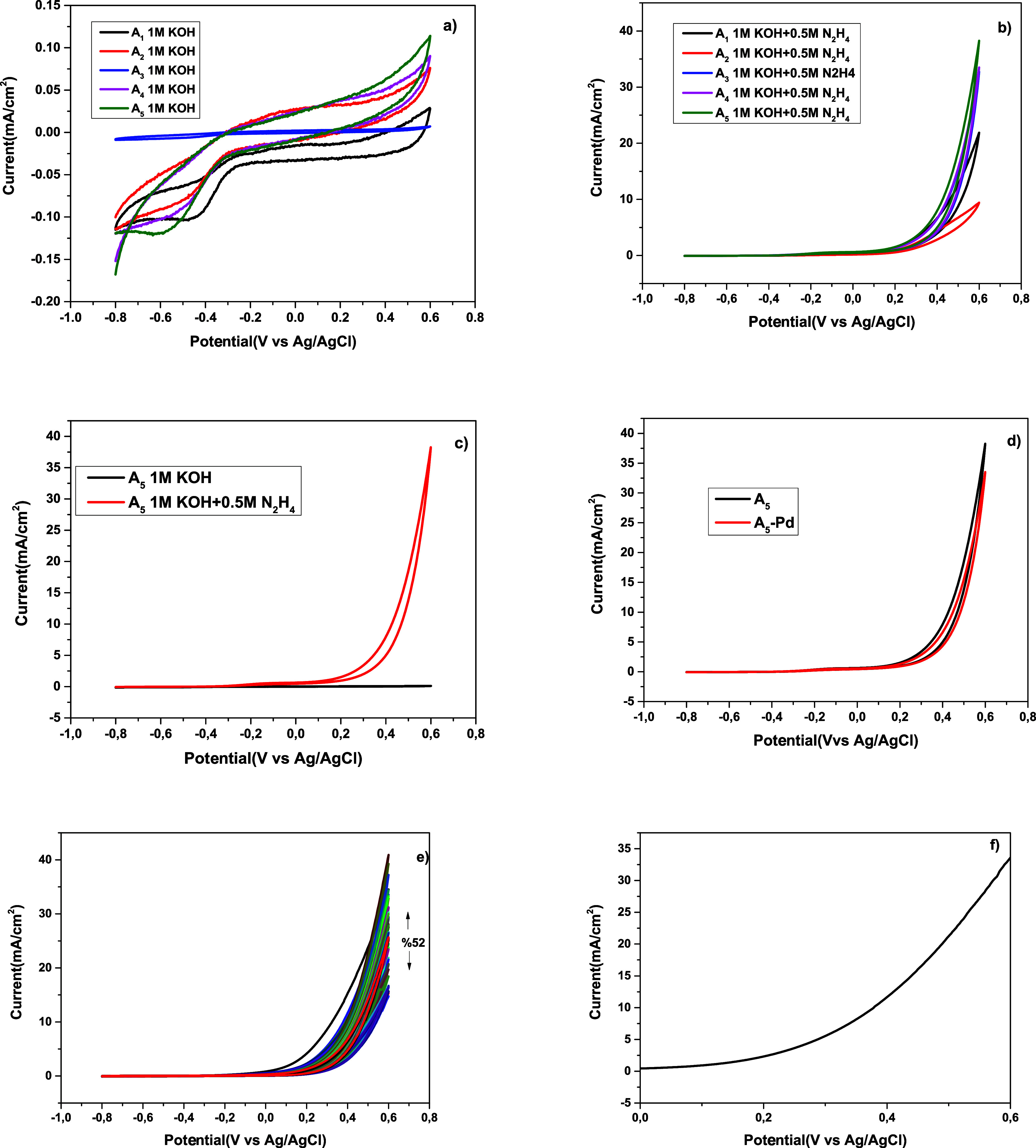
CV results of organocatalysts **A**_1_–**A**_5_ in a) 1 M KOH and b) 1 M KOH + 0.5 M N_2_H_4_, c) CV results of the dihydrobenzothienopyranone catalyst **A_5_** in 1 M KOH and 1 M KOH + 1 M KOH + 0.5 M N_2_H_4_, d) comparison of **A_5_**-Pd with **A_5_** catalyst in KOH (1 M) + N_2_H_4_ (0.5 M), e) consecutive CVs on catalyst **A**_**5**_ in 1 M KOH + 0.5 M N_2_H_4_ solution, and f) LSV measurement of **A**_**5**_ catalyst.

The hybrid **A_5_** organic-based catalyst’s electrochemical activity is investigated in Figure 2a at different scan rates between 10 and 1000 mV/s in a solution of 1 M KOH + 0.5 M N_2_H_4_. According to the findings, there is a high association between scan speed and current response, as the catalytic activity rises with scan rate. A linear association between the current values and the square root of the scan rate indicates that N_2_H_4_ electrooxidation belongs to a diffusion-controlled kinetics process, where the rate of reaction is dependent on the process of N_2_H_4_ diffusion onto the electrode surface. This again confirms the applicability of the hybrid catalyst **A**_**5**_ as an effective electrocatalyst for hydrazine electrooxidation. In Figure 2b, CV measurements were performed in 1 M KOH + 0.5 M N_2_H_4_ at scan rates ranging from 10 to 1000 mV s^–1^ to evaluate the electroactive surface area of the electrode. The Randles–Sevcik equation was used to determine the effective surface area, taking into account the solution concentration, the square root of the scan rate, and the number of electrons. As a result, an effective surface area with an *R* ^2^ value of 0.96 was observed.

**Figure 2:**
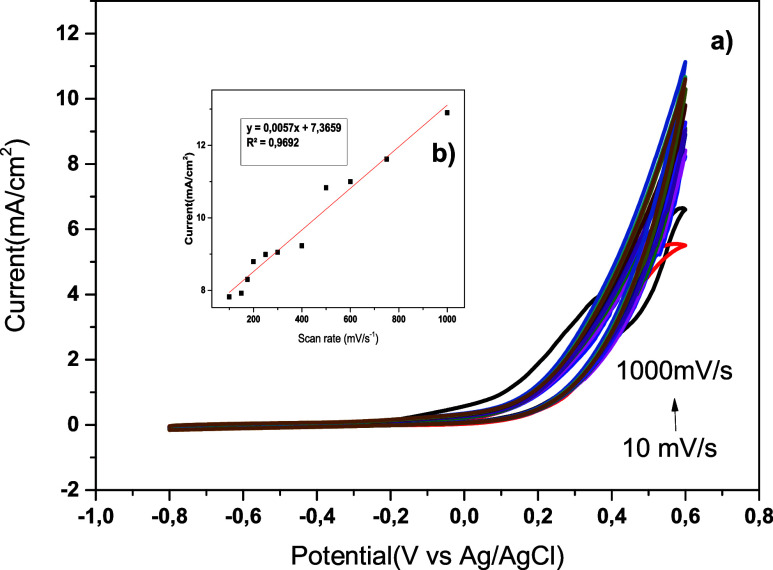
a) CV study of the hybrid dihydrobenzothienopyranone catalyst **A_5_** with varying scan rates (10–1000 mV/s), b) current versus scan rate graphs.

Chronoamperometry was used to investigate the stability of hybrid organic catalyst **A**_**5**_ and its resistance to poisoning. Figure 3 shows the CA curves for hybrid catalyst **A**_**5**_ at different potentials. The data indicate that this catalyst exhibits maximum stability and poisoning resistance at a potential of −0.6 V. This is the response that exists at this potential, which does not vary with time, demonstrating that the catalyst is indeed robust and can sustain activity under continuous electrochemical conditions. Such remarkable stability of the hybrid organic catalyst **A**_**5**_ in N_2_H_4_ electrooxidation makes it a very interesting option for long-term applications in fuel cells.

**Figure 3:**
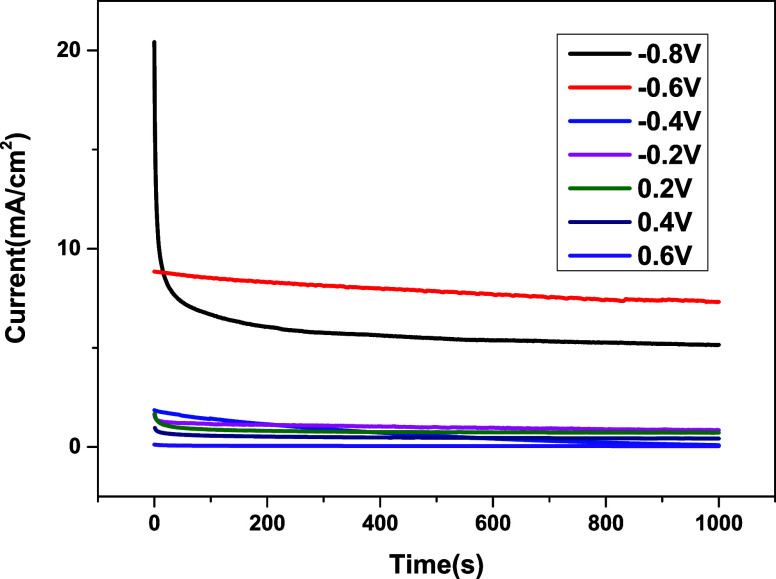
CA analysis of the hybrid dihydrobenzothienopyranone catalyst **A_5_**.

Figure 4a and b presents Nyquist plots of EIS conducted for the study of the electrocatalytic resistance of the organic-based catalyst **A**_**5**_. In general, electrochemical resistivity appears in the semicircular shape of the Nyquist plot during the EIS experiment due to the inverse relationship between the diameter-size and increasing resistance.^10,37^ Figure 4a displays the Nyquist plots for catalyst **A**_**5**_, which were obtained at different potentials between −0.8 and 0.6 V. At a voltage of 0.2 V, where the semicircle was largest and the impedance was highest, the highest electrochemical resistance was detected. The **A**_**5**_ organic-based catalyst’s charge transfer resistance (R_ct_) values, as determined in a solution of 1 M KOH + 0.5 M N_2_H_4_, are shown in Figure 4b. The R_ct_ values at the various potentials were 0.6 V (135.6 Ω), 0 V (123.2 Ω), 0.4 V (103.3 Ω), 0.2 V (92.47 Ω), and −0.2 V (89.41 Ω). The shortest semicircle in the Nyquist plot, represented by the lowest R_ct_ value at −0.2 V, indicated the maximum charge transfer efficiency at this potential. As a result, the dihydrobenzothienopyranone organic-based **A**_**5**_ catalyst’s superior electrocatalytic properties for N_2_H_4_ electrooxidation were highlighted when it showed the best carrier transfer performance at −0.2 V in comparison to the other potentials evaluated.

**Figure 4:**
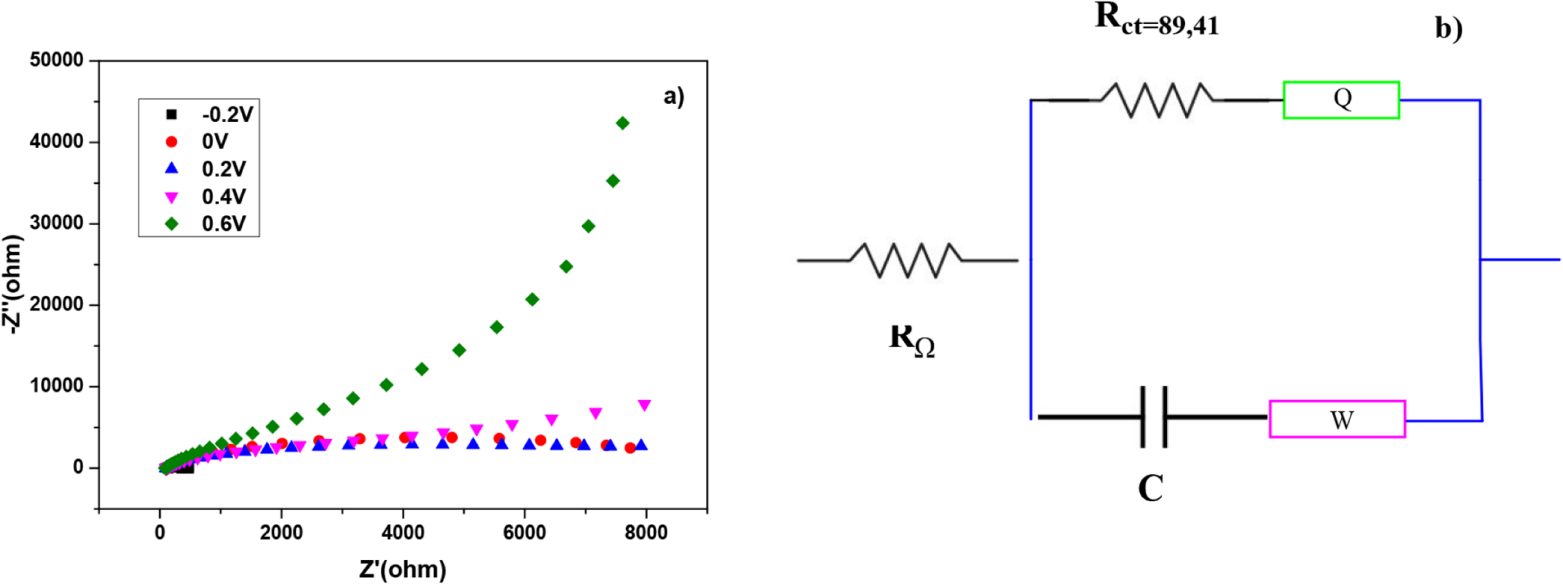
a) Nyquist graphs obtained for the dihydrobenzothienopyranone organic catalyst **A_5_** in 1 M KOH + 0.5 M N_2_H_4_ at different potentials. b) R_ct_ value.

The possible reaction mechanism is shown in Figure 5. The mechanism begins with the formation of a hydrogen bond between hydrazine monohydrate and the carbonyl oxygen of **A**_**5**_, assisted by additional weak interactions with the heteroatoms in the furan–benzothienopyran scaffold. This interaction facilitates the dehydration of hydrazine monohydrate, generating an activated hydrazine species on the catalyst surface. In the next step, the base present in the electrolyte abstracts protons from hydrazine, yielding intermediate species such as N_2_H_3_ **·** and N_2_H_2_. The conjugated π-system of **A**_**5**_ stabilizes these intermediates and promotes stepwise electron transfer. Ultimately, a total of four electrons are released, and molecular nitrogen is formed and desorbed from the catalyst surface, regenerating **A**_**5**_ for the next catalytic cycle.

**Figure 5:**
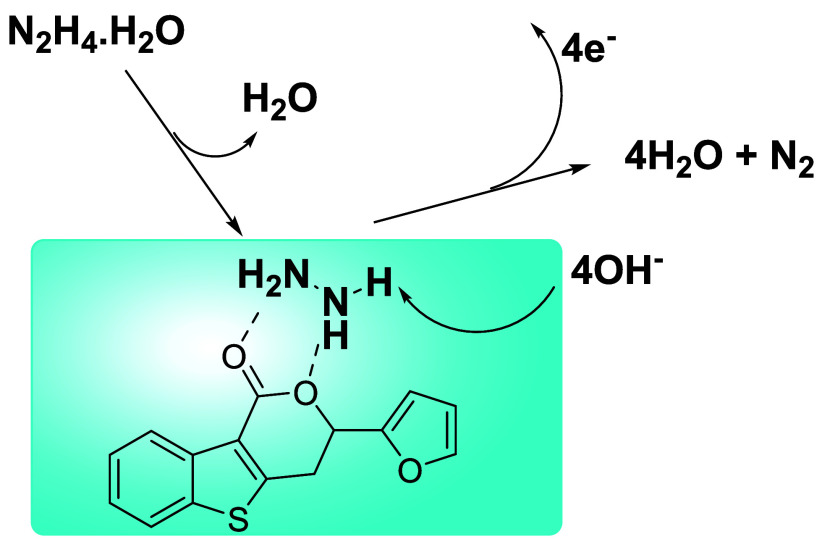
Proposed mechanism for hydrazine electrooxidation catalyzed by **A**_**5**_.

## 4 Conclusions

This study presents the design, synthesis, and comprehensive electrochemical evaluation of five dihydrobenzothienopyranone derivatives (**A**_**1**_–**A**_**5**_) as anode catalysts for the electrooxidation of N_2_H_4_. According to the experimental findings, 3-(furan-2-yl)-3,4-dihydro-1*H*-benzo­[4,5]-thieno­[3,2-*c*]­pyran-1-one **A**_**5**_ among these compounds, shows the most promising catalytic activity, with a current density of 38.29 mA/cm^2^, as well as exceptional stability and electrochemical performance. 3-(Furan-2-yl)-3,4-dihydro-1*H*-benzo­[4,5]-thieno­[3,2-*c*]­pyran-1-one catalyst **A**_**5**_ may prove to be a viable and economical substitute for conventional precious metal-based catalysts, such as palladium and platinum, which are frequently employed in fuel cell applications, according to these exceptional properties. Since N_2_H_4_ is relatively inexpensive and has a higher energy density, the results pave the way for the creation of direct hydrazine fuel cells, which are thought to be a more sustainable energy source. The true commercial potential of these organic-based catalysts will also depend on their integration into workable fuel cell systems and the additional optimization of their production, structure, and electrochemical performance. These developments might play a big role in the near future shift to fuel cell technologies that are more affordable, ecologically friendly, and efficient.

## Supplementary Material



## Notes

Additional experimental results; intermediates and final compounds, ^1^H and ^13^C NMR spectra; and representative HRMS spectra of the final products (PDF)

## References

[1] Bakr R. A., Ulas B., Kivrak H. (2017). A mini review
on health and environmental
risks of oil and gas industry undesired products: Hydrogen sulfide
and Carbon monoxide. Int. J. Ecosyst. Ecol.
Sci..

[2] Muthukumar V., Chetty R. (2017). Morphological transformation
of electrodeposited Pt
and its electrocatalytic activity towards direct formic acid fuel
cells. J. Appl. Electrochem..

[3] Kivrak H., Atbas D., Alal O., Çögenli M. S., Bayrakceken A., Mert S. O., Sahin O. (2018). A complementary study
on novel PdAuCo catalysts: Synthesis, characterization, direct formic
acid fuel cell application, and exergy analysis. Int. J. Hydrogen Energy.

[4] Caglar A., Sahan T., Cogenli M. S., Yurtcan A. B., Aktas N., Kivrak H. (2018). A novel central composite
design based response surface
methodology optimization study for the synthesis of Pd/CNT direct
formic acid fuel cell anode catalyst. Int. J.
Hydrogen Energy.

[5] Bulut A., Yurderi M., Karatas Y., Say Z., Kivrak H., Kaya M., Gulcan M., Ozensoy E., Zahmakiran M. (2015). MnO x-promoted
PdAg alloy nanoparticles for the additive-free dehydrogenation of
formic acid at room temperature. ACS Catal..

[6] Feng G., Kuang Y., Li P., Han N., Sun M., Zhang G., Sun X. (2017). Single crystalline
ultrathin nickel–cobalt
alloy nanosheets array for direct hydrazine fuel cells. Adv. Sci..

[7] Er O. F., Caglar A., Kivrak H. (2020). Enhanced electrochemical
glucose
oxidation in alkaline solution over indium decorated carbon supported
palladium nanoparticles. Mater. Chem. Phys..

[8] Ulas B., Caglar A., Kivrak A., Kivrak H. (2019). Atomic molar ratio
optimization of carbon nanotube supported PdAuCo catalysts for ethylene
glycol and methanol electrooxidation in alkaline media. Chem. Pap..

[9] Kivrak, H. ; Kuliyev, S. ; Tempel, H. ; Schneider, J. ; Uner, D. Carbon nanotube structures as support for ethanol electro-oxidation catalysis. Int. J. Chem. React. Eng. 2011. 9 1. 10.1515/1542-6580.2437

[10] Ulas B., Caglar A., Sahin O., Kivrak H. (2018). Composition dependent
activity of PdAgNi alloy catalysts for formic acid electrooxidation. J. Colloid Interfaces Sci..

[11] Kaya S., Caglar A., Kivrak H. (2022). Carbon nanotube
supported Ga@ PdAgCo
anode catalysts for hydrazine electrooxidation in alkaline media. Fuel.

[12] Ding J., Wang H. F., Yang X., Ju W., Shen K., Chen L., Li Y. (2023). A Janus heteroatom-doped
carbon electrocatalyst
for hydrazine oxidation. Nat. Sci. Rev..

[13] Askari M. B., Salarizadeh P., Beitollahi H., Tajik S., Eshghi A., Azizi S. (2022). Electro-oxidation of
hydrazine on NiFe_2_O_4_-rGO
as a high-performance nano-electrocatalyst in alkaline media. Mater. Chem. Phys..

[14] Er O. F., Ulas B., Ozok O., Kivrak A., Kivrak H. (2021). Design of
2-(4-(2-pentyllbenzo [b] thiophen-3-yl) benzylidene) malononitrile
based remarkable organic catalyst towards hydrazine electrooxidation. J. Electroanal. Chem..

[15] Zabielaitė A., Balčiu̅naitė A., Šimku̅naitė D., Lichušina S., Stalnionienė I., Šimku̅naitė-Stanynienė B., Naruškevičius L., Tamašauskaitė-Tamašiu̅naitė L., Norkus E., Selskis A. (2020). High performance direct N_2_H_4_-H_2_O_2_ fuel cell using fiber-shaped
Co decorated with Pt crystallites as anode electrocatalysts. J. Electrochem. Soc..

[16] Liang B., Wang Y., Liu X., Tan T., Zhang L., Wang W. (2019). Nickel–cobalt alloy doping phosphorus as advanced electrocatalyst
for hydrazine oxidation. J. Alloys Compd..

[17] Burshtein T. Y., Farber E. M., Ojha K., Eisenberg D. (2019). Revealing
structure–activity links in hydrazine oxidation: doping and
nanostructure in carbide–carbon electrocatalysts. J. Mater. Chem. A.

[18] Yi Q., Chu H., Tang M., Zhang Y., Liu X., Zhou Z., Nie H. (2014). A novel membraneless
direct hydrazine/air fuel cell. Fuel Cells.

[19] Raoof J. B., Ojani R., Jamali F., Hosseini S. R. (2012). Electrochemical
detection of hydrazine using a copper oxide nanoparticle modified
glassy carbon electrode. Caspian J. Chem..

[20] Jia F., Zhao J., Yu X. (2013). Nanoporous
Cu film/Cu plate with
superior catalytic performance toward electro-oxidation of hydrazine. J. Power Sources.

[21] Yan X., Meng F., Xie Y., Liu J., Ding Y. (2012). Direct N2H4/H2O2
fuel cells powered by nanoporous gold leaves. Sci. Rep..

[22] Asset T., Roy A., Sakamoto T., Padilla M., Matanovic I., Artyushkova K., Serov A., Maillard F., Chatenet M., Asazawa K. (2016). Highly active and selective nickel molybdenum catalysts
for direct hydrazine fuel cell. Electrochim.
Acta.

[23] Lin X., Wen H., Zhang D. X., Cao G. X., Wang P. (2020). Highly dispersed nickel
nitride nanoparticles on nickel nanosheets as an active catalyst for
hydrazine electrooxidation. J. Mater. Chem.
A.

[24] Meng Y., Zou X., Huang X., Goswami A., Liu Z., Asefa T. (2014). Polypyrrole-derived
nitrogen and oxygen co-doped mesoporous carbons as efficient metal-free
electrocatalyst for hydrazine oxidation. Adv.
Mater..

[25] Wang Y., Wang Q., Wan L. Y., Han Y., Hong Y., Huang L., Yang X., Wang Y., Zaghib K., Zhou Z. (2020). KOH-doped polybenzimidazole membrane for direct hydrazine fuel cell. J. Colloid Interfaces Sci..

[26] Wen H., Cao G. X., Chen M. H., Qiu Y. P., Gan L. Y., Wang P. (2020). Surface phosphorization
of hierarchically nanostructured nickel molybdenum
oxide derived electrocatalyst for direct hydrazine fuel cell. Appl. Catal., B.

[27] Hama A. K., Algso M. A. S., Kavak E., Kivrak A. R. İ. F. (2020). Synthesis
of novel benzothiophene derivatives via cyclization reactions. Russ. J. Org. Chem..

[28] Carbas B. B., Kivrak A., Teke E., Zora M., Önal A. M. (2014). Electrochemical
polymerization of a new low-voltage oxidized thienylenepyrrole derivative
and its electrochromic device application. J.
Electroanal. Chem..

[29] Kivrak A., Carbas B. B., Zora M., Önal A. M. (2012). Synthesis
and electropolymerization of an ion sensing and fluorescent fluorene
derivative bearing a quinoxaline moiety and its analogues with different
donor units. React. Funct. Polym..

[30] Karatekin H. C., Ulas B., Yilmaz Y., Kivrak H., Kivrak A. (2024). Hydrazine
electrooxidation performance of cyano-substituted indole derivatives
as organic anode catalyst. Process Saf. Environ.
Prot..

[31] Chen L. X., Jiang L. Y., Wang A. J., Chen Q. Y., Feng J. J. (2016). Simple
synthesis of bimetallic AuPd dendritic alloyed nanocrystals with enhanced
electrocatalytic performance for hydrazine oxidation reaction. Electrochim. Acta.

[32] Liu R., Yin J., Cao D. (2017). Ti Foil Supported
Three-Dimensional Porous Silver Film
as a High Performance Catalyst for Hydrazine Electrooxidation in KOH
Solution. Int. J. Electrochem. Sci..

[33] Sharif K. H., Kivrak H., Ozok-Arici O., Caglar A., Kivrak A. (2022). Catalytic
electro-oxidation of hydrazine by thymol based-modified glassy carbon
electrode. Fuel.

[34] Ziccarelli I., Mancuso R., Santandrea D., Altomare A., Olivieri D., Carfagna C., Gabriele B. (2023). Synthesis
of benzothienofuranones
and dihydrobenzothienopyranones by palladium iodide catalyzed carbonylative
double cyclization. J. Catal..

[35] Wang J., Sun H. B., Shah S. A., Liu C., Zhang G. Y., Li Z., Zhang Q. F., Han M. (2020). Palladium
nanoparticles supported
by three-dimensional freestanding electrodes for high-performance
methanol electro-oxidation. Int. J. Hydrogen
Energy.

[36] Ulaş B. (2023). Response surface
methodology optimization of electrode modification parameters toward
hydrazine electrooxidation on Pd/MWCNT/GCE. MANAS J. Eng..

[37] Hansu T. A., Caglar A., Sahin O., Kivrak H. (2020). Hydrolysis and electrooxidation
of sodium borohydride on novel CNT supported CoBi fuel cell catalyst. Mater. Chem. Phys..

