# Supplementary material for: Investigation of Hydrazine Electrooxidation Performance of Dihydrobenzothienopyranone Derivatives
Source: ACS Omega. 2026 Mar 11;11(11):17900–6. doi: 10.1021/acsomega.5c12506 (PMC13019249; doi:10.1021/acsomega.5c12506)
Supplement: Supplementary file 1 [file ao5c12506_si_001.pdf]

Supporting Information for

Investigation of hydrazine electrooxidation performance of dihydrobenzothienopyranone derivatives

Omruye Ozok Arıcı[1], Raffaella Mancuso[2], Bartolo Gabriele[2], Hilal Kivrak[3], Arif Kivrak[4*]

[1]Department of Biomedical Engineering, Faculty of Engineering and Architectural Sciences, Eskisehir Osmangazi University, Eskisehir, Turkey

[2]Laboratory of Industrial and Synthetic Organic Chemistry (LISOC), Department of Chemistry and Chemical Technologies, University of Calabria, Via Pietro Bucci 12/C, 87036 Arcavacata di Rende (CS), Italy

[3]Department of Chemical Engineering, Faculty of Engineering and Architectural Sciences, Eskisehir Osmangazi University, Eskisehir, Turkey

[4]Department of Chemistry, Faculty of Sciences and Arts, Eskisehir Osmangazi University, Eskişehir, Turkey

**Corresponding Author: arif.kivrak@ogu.edu.tr*

Table of Contents

Pages S2–S3 **Preparation and characterization of 4-(2-(methylthio)phenyl)but-3-yn-1-ols B_1-B_5.**

Pages S4–S5 **General procedure for the synthesis of benzo[4,5]thieno[3,2-c]pyran-1-ones A_1-A_5 from 4-(2-(methylthio)phenyl)but-3-yn-1-ols B_1-B_5, and characterization.**

Page S5 **References**

Pages S6−S9 **Copies of HRMS spectra**

Pages S10−S29 **Copies of ^{1}H NMR and ^{13}CNMR spectra**

Preparation and characterization of 4-(2-(methylthio)phenyl)but-3-yn-1-ols B_1-B_5.[1]

Synthesis of 4-(2-(methylthio)phenyl)but-3-yn-1-ols B_1-B_5.

4-(2-(Methylthio)phenyl)but-3-yn-1-ols **3** were prepared by Sonogashira coupling of 2-iodothioanisole (commercially available), 2-bromo-5-fluorothioanisole (prepared by methylation of commercially available 2-bromo-5-fluorobenzenethiol, according to a literature procedure [2]) with but-3-yn-1-ols, as described below. But-3-yn-1-ol, and hex-5-yn-3-ol are commercially available; 1-phenylbut-3-yn-1-ol, 1-(furan-2-yl)but-3-yn-1-ol, 2-phenylpent-4-yn-2-ol were prepared by Barbier reaction between an alkynyl bromide and an aldehyde or a ketone in the presence of activated zinc [3].

General procedure for the synthesis of 4-(2-(methylthio)phenyl)but-3-yn-1-ols B_1, B_3-B_5.

A solution of 2-iodothioanisole (500 mg, 2.0 mmol), $PdCl_2(PPh_3)_2$ (28 mg, 0.04 mmol), CuI (11.4 mg, 0.06 mmol), and the terminal alkyne (2.4 mmol; but-3-yn-1-ol, 168 mg; pent-4-yn-2-ol, 202 mg; hex-5-yn-3-ol, 235 mg; 1-phenylbut-3-yn-1-ol, 350 mg; 1-mesitylbut-3-yn-1-ol, 452 mg; 1-(4-methoxyphenyl)but-3-yn-1-ol, 422 mg; 1-(furan-2-yl)but-3-yn-1-ol, 327 mg; 2-phenylpent-4-yn-2-ol, 385 mg; 1-(prop-2-yn-1-yl)cyclohexan-1-ol, 332 mg; *trans*-2-ethynylcyclohexan-1-ol, 300 mg; *trans*-2-ethynylcyclopentan-1-ol, 265 mg) in anhydrous triethylamine (8 mL) was allowed to stir under nitrogen at 25 °C for 15 h. The mixture was washed with a saturated solution of NH_4Cl (3 x 20 mL); the aqueous layer was extracted with ethyl acetate (3 x 20 mL), and the combined organic layers was washed with a saturated solution of NH_4Cl until pH was neutral, then dried with Na_2SO_4, and concentrated in vacuo. The resulting crude product was purified via chromatography on silica gel using 9:1 hexane–AcOEt to 6:4 hexane–AcOEt as eluent.

6-(2-(Methylthio)phenyl)hex-5-yn-3-ol (**B_1**). Yield: 430 mg, starting from 500 mg of 2-iodothioanisole (98%). Yellow oil. IR (film): ν = 3410 (m, br), 2222 (w), 1582 (w), 1466 (m), 1435 (s), 1111 (m), 1072 (w), 1081 (w), 980 (m), 748 (s) cm^{-1}; ^1H NMR ($CDCl_3$, 500 MHz): δ = 7.36 (dist dd, *J* = 7.6, 1.3, 1H, H-6), 7.27 (dist dd, *J* = 7.6, 1.3, 1H, H-4), 7.14 (dist d, *J* = 7.9, 1H, H-3), 7.06 (td, *J* = 7.6, 0.9, 1H, H-5), 3.84-3.76 (m, 1H, C*H*OH), 2.72 (dist dd, *J* = 16.7, 4.5, 1H, ≡CC*H*H), 2.60 (dist dd, *J* = 16.7, 7.0, 1H, ≡CCH*H*), 2.47 (s, 3H, SMe), 2.48-2.44 (m, 1H, OH), 1.71 -1.62 (m, 2H, CH_2CH_3), 1.00 (t, *J* = 7.4, 3H, CH_2CH_3); ^{13}C-NMR ($CDCl_3$, 125 MHz): δ = 141.3, 132.0, 128.5, 124.3, 124.1, 121.6, 93.3, 80.8, 71.6, 29.2, 28.3, 15.1, 10.1; GC-MS (EI, 70 eV) *m/z* = 220 (M^+, 8), 187 (7), 162 (26), 147 (100), 128 (14), 115 (23); HRMS-ESI (*m/z*): [$(M+H)^+$] cald for $(C_{13}H_{17}OS)^+$: 221.0994; found, 221.1004.

5-(2-(Methylthio)phenyl)-2-phenylpent-4-yn-2-ol (**B_3**). Yield: 453 mg, starting from 500 mg of 2-iodothioanisole (80%). Yellow oil. IR (film): ν = 3443 (m, br), 2232 (vw), 1582 (w), 1493 (m), 1464 (m), 1435 (w), 1180 (w), 1099 (m), 1028 (m), 912 (m), 766 (m), 719 (s), 700 (s) cm^{-1}; ^1H NMR ($CDCl_3$, 500 MHz): δ = 7.57-7.52 (m, 2H, aromatic), 7.37-7.32 (m, 2H, aromatic), 7.31 (dd, *J* = 7.6, 1.3, 1H, aromatic), 7.28-7.22 (m, 2H, aromatic), 7.13 (dist d, *J* = 8.6, 1H, H-3), 7.04 (dist td, *J* = 7.5, 1.0, 1H, H-5), 3.04 (dist d, *J* = 16.7, 1H, ≡CC*H*H), 2.97 (dist d, *J* = 16.7, 1H, ≡CCH*H*), 2.93 (s, 1H, OH), 2.43 (s, 3H, SMe), 1.73 (s, 3H, Me); ^{13}C-NMR ($CDCl_3$, 125 MHz): δ = 146.6, 141.4, 132.1, 128.5, 128.2, 127.0, 124.8, 124.4, 121.6, 92.7, 81.7, 73.6, 36.0, 29.5, 15.3; GC-MS (EI, 70 eV): *m/z* = 282 (M^+, 2), 263 (54), 248 (44), 234 (72), 215 (26), 202 (16), 162 (60), 147 (100), 121 (8), 115 (47), 105 (41); HRMS-ESI (*m/z*): [$(M-H_2O+H)^+$] calcd for $(C_{18}H_{17}S)^+$: 265.1045; found, 265.1053.

4-(2-(Methylthio)phenyl)-1-phenylbut-3-yn-1-ol (**B_4**). Yield: 375 mg, starting from 500 mg of 2-iodothioanisole (70%). Yellow oil. IR (film): ν = 3418 (m, br), 2230 (vw), 1582 (m), 1458 (m), 1435 (m), 1196 (w), 1042 (m), 748 (s), 702 (m) cm^{-1}; ^1H NMR ($CDCl_3$, 500 MHz): δ = 7.48-7.42 (m, 2H, aromatic), 7.39-7.32 (m, 3H, aromatic), 7.32-7.23 (m, 2H, aromatic), 7.14 (dist d, *J* = 7.9, 1H, H-3), 7.06 (td, *J* = 7.5, 0.9, 1H, H-5), 5.02-4.92 (m, 1H, C*H*OH), 2.99-2.91 (m, 2H, ≡CC*H*H + OH), 2.89 (dist dd, *J* = 16.7, 7.8, 1H, ≡CCH*H*), 2.46 (s, 3H, SMe); ^{13}C-NMR ($CDCl_3$, 125 MHz): δ = 142.6, 141.4, 132.1, 128.6, 128.5, 127.8, 125.8, 124.4,

124.3, 121.5, 93.0, 88.1, 72.5, 31.1, 15.2; GC-MS (EI, 70 eV): *m/z* = 268 (M^+, 5), 267 (13), 235 (7), 162 (20), 147 (100), 128 (11), 115 (12), 107 (22), 79 (41); HRMS-ESI (*m/z*): [$(M-H_2O+H)^+$] calcd for $(C_{17}H_{15}S)^+$: 251.0889; found, 251.0880.

1-(Furan-2-yl)-4-(2-(methylthio)phenyl)but-3-yn-1-ol (**B$_5$**). Yield: 488 mg, starting from 500 mg of 2-iodothioanisole (94%). Yellow oil. IR (film): ν = 3418 (m, br), 2230 (vw), 1582 (w), 1504 (w), 1466 (w), 1435 (m), 1227 (w), 1142 (w), 1042 (m), 1011 (m), 748 (s) cm^{-1}; ^{1}H NMR ($CDCl_3$, 500 MHz): δ = 7.41-7.38 (m, 1H, furyl ring), 7.35 (dd, *J* = 7.6, 1.2, 1H, H-6), 7.29-7.24 (m, 1H, H-4), 7.14 (dist d, *J* = 8.0, 1H, H-3), 7.06 (td, *J* = 7.6, 1.0, 1H, H-5), 64.1-6.39 (m, 1H, furyl ring), 6.36-6.33 (m, 1H, furyl ring), 5.00 (q, *J* = 5.9, 1H, C*H*OH), 3.06 (dist d, *J* = 5.9, 2H, ≡CCH_2), 2.92 (d, *J* = 5.9, 1H, OH), 2.46 (s, 3H, SMe); ^{13}C-NMR ($CDCl_3$, 125 MHz): δ = 154.8, 142.2, 141.4, 132.1, 128.6, 124.39, 124.38, 121.5, 110.3, 106.8, 92.1, 81.3, 66.4, 27.6, 15.3; GC-MS (EI, 70 eV): *m/z* = 258 (M^+, 2), 243 (33), 197 (5), 162 (32), 147 (100), 115 (13), 97 (84); HRMS-ESI (*m/z*): [$(M-H_2O+H)^+$] calcd for $(C_{15}H_{13}OS)^+$: 241.0681; found, 241.0690.

Procedure for the synthesis of 4-(4-fluoro-2-(methylthio)phenyl)but-3-yn-1-ol B$_2$.

A solution of 2-bromo-5-fluorothioanisole (440 mg, 2.0 mmol), $PdCl_2(PPh_3)_2$ (140 mg, 0.2 mmol), CuI (57 mg, 0.3 mmol), and but-3-yn-1-ol (280.4 mg, 4.0 mmol) in anhydrous diisopropylamine (20 mL) was allowed to stir under nitrogen at 80 °C for 24 h. Water (50 mL) was then added, and the mixture extracted with diethyl ether (3 × 50 mL). The organic layer was washed with a saturated solution of NH_4Cl (100 mL) and water until neutral pH. After drying over Na_2SO_4, the solvent was evaporated, and the residue purified by column chromatography on silica gel using 9:1 hexane–AcOEt to 6:4 hexane–AcOEt as eluent.

4-(4-Fluoro-2-(methylthio)phenyl)but-3-yn-1-ol (**B$_2$**). Yield: 307 mg, starting from 440 mg of 2-bromo-5-fluorothioanisole (73%). Yellow oil. IR (film): ν = 3397 (m, br), 2230 (w), 1591 (m), 1568 (w), 1477 (s), 1435 (m), 1250 (m), 1202 (m), 1044 (s), 897 (w), 847 (m) cm^{-1}; ^{1}H NMR ($CDCl_3$, 500 MHz): δ = 7.32 (dd, *J* = 8.4, 5.9, 1H, H-6), 6.82 (dd, *J* = 9.7, 2.5, 1H, H-3), 6.75 (td, *J* = 8.4, 2.5, 1H, H-5), 3.84 (q, *J* = 6.1, 2H, C*H*$_2$OH), 2.74 (t, *J* = 6.1, 2H, C*H*$_2$CH$_2$OH), 2.46 (s, 3H, SMe), 2.27-2.21 (m, 1H, OH); ^{13}C-NMR ($CDCl_3$, 125 MHz): δ = 162.7 (d, *J* = 250.2), 144.3 (d, *J* = 8.4), 133.5 (d, *J* = 8.9), 117.1 (d, *J* = 2.9), 113.3 (d, *J* = 22.2), 111.0 (d, *J* = 24.8), 93.2, 79.3, 61.1, 24.1, 14.9 (d, *J* = 3.1); GC-MS (EI, 70 eV) *m/z* = 210 (M^+, 61), 179 (12), 165 (100), 146 (55), 133 (36), 115 (33); HRMS-ESI (*m/z*): [$(M+H)^+$] cald for $(C_{11}H_{12}FOS)^+$: 211.0587; found: 211.0595.

General procedure for the synthesis of benzo[4,5]thieno[3,2-c]pyran-1-ones A_1-A_5 from 4-(2-(methylthio)phenyl)but-3-yn-1-ols B_1-B_5 and characterization.[1]

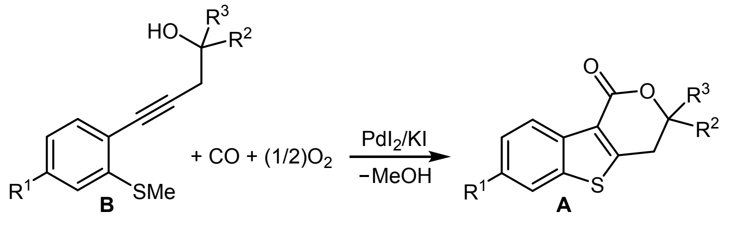

A 50 mL stainless steel autoclave was charged in the presence of air with PdI_2 (25.0 mg, 6.9 ×10^{-2} mmol), KI (802 mg, 4.83 mmol) and a solution of **B** (0.69 mmol; $\mathbf{B_1}$, 152 mg; $\mathbf{B_2}$, 145 mg; $\mathbf{B_3}$, 195 mg; $\mathbf{B_4}$, 186 mg; $\mathbf{B_5}$, 178 mg) in MeCN (6.9 mL). The autoclave was sealed and, while the mixture was stirred, the autoclave was pressurized with CO (48 atm) and air (12 atm). After being stirred at 80 °C for 3 h ($\mathbf{B_1}$-$\mathbf{B_2}$, $\mathbf{B_4}$-$\mathbf{B_5}$), or 15 h ($\mathbf{B_3}$), the autoclave was cooled, degassed and opened. The solvent was evaporated, and products $\mathbf{A_1}$-$\mathbf{A_5}$ were purified by column chromatography on silica gel using as eluent 98:2 hexane-AcOEt to 9:1 hexane-AcOEt.

3-Ethyl-3,4-dihydro-1H-benzo[4,5]thieno[3,2-c]pyran-1-one ($\mathbf{A_1}$). Yield: 112 mg, starting from 152 mg of 6-(2-(methylthio)phenyl)hex-5-yn-3-ol $\mathbf{B_1}$ (70%; Table 3, entry 7). White solid, mp = 139-140 °C. IR (KBr): ν = 1713 (s), 1528 (w), 1466 (m), 1396 (m), 1204 (s), 1111 (m), 1065 (w), 972 (w), 787 (m) cm^{-1}; ^{1}H NMR (500 MHz, $CDCl_3$): δ = 8.52 (d, *J* = 8.0, 1H, H-8), 7.79 (d, *J* = 8.1, 1H, H-5), 7.51-7.45 (m, 1H, H-6 or H-7), 7.43-7.35 (m, 1H, H-7 or H-6), 4.63-4.56 (m, 1H, OC*H*CH_2), 3.14 (dist dd, *J* = 17.1, 4.9, 1H, C*H*HCHEt), 3.09 (dist dd, *J* = 17.1, 10.5, 1H, CH*H*CHEt), 2.03-1.92 (m, 1H, C*H*HCH_3), 1.90-1.80 (m, 1H, CH*H*CH_3), 1.12 (t, *J* = 7.5, 3 H, CH_2CH_3); ^{13}C NMR (125 MHz, $CDCl_3$): δ = 161.3, 152.6, 137.8, 136.4, 125.9, 125.4, 124.5, 122.1, 121.5, 80.0, 30.0, 27.9, 9.5; GC-MS: *m/z* = 232 (M$^+$, 54), 203 (5), 174 (100), 146 (68), 102 (30); HRMS-ESI (*m/z*): [(M+H)$^+$] calcd for $(C_{13}H_{13}O_2S)^+$: 233.0630; found, 233.0615.

7-Fluoro-3,4-Dihydro-1H-benzo[4,5]thieno[3,2-c]pyran-1-one ($\mathbf{A_2}$). Yield: 85 mg, starting from 145 mg of 4-(4-fluoro-2-(methylthio)phenyl)but-3-yn-1-ol $\mathbf{B_2}$ (55%; Table 3, entry 5). Pale yellow solid, mp = 176-178 °C. IR (KBr): ν = 1707 (s), 1535 (w), 1481 (m), 1341 (w), 1267 (m), 1184 (m), 1064 (m), 995 (w), 822 (m), 772 (s) cm^{-1}; ^{1}H NMR (500 MHz, $CDCl_3$): δ = 8.46 (dd, *J* = 8.9, 5.3, H-8), 7.49 (dd, *J* = 8.6, 2.3, 1H, H-5), 7.23 (td, *J* = 8.9, 2.3, 1H, H-7), 4.66 (t, *J* = 6.2, 2H, OCH_2), 3.25 (t, *J* = 6.2, 2H, OCH_2CH_2); ^{13}C NMR (125 MHz, $CDCl_3$): δ = 160.9 (d, *J* = 246.4), 160.7, 152.2 (d, *J* = 2.8), 138.6 (d, *J* = 10.2), 132.9, 125.8 (d, *J* = 8.9), 121.2, 114.9 (d, *J* = 23.6), 108.5 (d, *J* = 25.7), 67.2, 25.4; GC-MS: *m/z* = 222 (M$^+$, 94), 192 (67), 164 (100), 133 (9), 120 (53), 96 (24); HRMS-ESI (*m/z*): [(M+H)$^+$] calcd for $(C_{11}H_8FO_2S)^+$: 223.0224; found, 223.0234.

3-Methyl-3-phenyl-3,4-dihydro-1H-benzo[4,5]thieno[3,2-c]pyran-1-one ($\mathbf{A_3}$). Yield: 161 mg, starting from 195 mg of 5-(2-(methylthio)phenyl)-2-phenylpent-4-yn-2-ol $\mathbf{B_3}$ (79%; Table 3, entry 12). White solid, mp = 137-138 °C. IR (KBr): ν = 1711 (s), 1466 (m), 1433 (w), 1383 (m), 1267 (w), 1219 (m), 1115 (m), 1063 (w), 993 (m), 766 (m) cm^{-1}; ^{1}H NMR (500 MHz, $CDCl_3$): δ = 8.48 (d, *J* = 8.0, 1H, H-8), 7.73 (d, *J* = 7.9, 1H, H-5), 7.49-7.39 (m, 3H, aromatic), 7.36-7.26 (m, 3H, aromatic), 7.23-7.17 (m, 1H), 3.70 (dist d, *J* = 17.2, 1H, C*H*H), 3.52 (dist d, *J* = 17.2, 4.0, 1H, CH*H*Ph), 1.81 (s, 3H, Me); ^{13}C NMR (125 MHz, $CDCl_3$): δ = 160.6, 151.6, 143.4, 137.6, 136.3, 128.7, 127.7, 125.8, 125.3, 124.43. 124.36, 122.0, 121.5, 84.3, 36.1, 30.0; GC-MS: *m/z* = 294 (M$^+$, 14), 249 (2), 234 (5), 174 (100), 146 (51), 102 (21), 77 (16); HRMS-ESI (*m/z*): [(M+H)$^+$] calcd for $(C_{18}H_{15}O_2S)^+$: 295.0787; found, 295.0797.

3-Phenyl-3,4-dihydro-1H-benzo[4,5]thieno[3,2-c]pyran-1-one ($\mathbf{A_4}$). Yield: 154 mg, starting from 186 mg of 4-(2-(methylthio)phenyl)-1-phenylbut-3-yn-1-ol $\mathbf{B_4}$ (79%; Table 3, entry 8). Pale yellow solid, mp = 167-168 °C. IR (KBr): ν = 1713 (s), 1466 (w), 1381 (m), 1265 (w), 1204 (m), 1119 (w), 1057 (w), 772 (m) cm^{-1}; ^{1}H NMR (500 MHz, $CDCl_3$): δ = 8.55 (d, *J* = 7.9, 1H, H-8), 7.81 (d, *J* = 8.0, 1H, H-5), 7.56-7.45 (m, 3H, aromatic), 7.45-7.34 (m, 4H, aromatic), 5.66 (dd, *J* = 11.5, 4.0, 1H, C*H*Ph), 3.42 (dist dd, *J* = 17.2, 11.5, 1H, C*H*HCHPh),

3.34 (dist dd, J = 17.2, 4.0, 1H, CH*H*Ph); ^{13}C NMR (125 MHz, $CDCl_3$): δ = 160.7, 152.4, 138.0, 137.8, 136.3, 128.83, 128.76, 126.2, 126.0, 125.5. 124.5, 122.1, 121.6, 80.0, 32.7; GC-MS: m/z = 280 (M^+, 18), 234 (6), 174 (100), 146 (70), 102 (31); HRMS-ESI (m/z): [$(M+H)^+$] calcd for $(C_{17}H_{13}O_2S)^+$: 281.0630; found, 281.0647.

3-(Furan-2-yl)-3,4-dihydro-1H-benzo[4,5]thieno[3,2-c]pyran-1-one (**A$_5$**). Yield: 112 mg, starting from 178 mg of 1-(furan-2-yl)-4-(2-(methylthio)phenyl)but-3-yn-1-ol **B$_5$** (60%; Table 3, entry 11). Pale yellow solid, mp = 140-142 °C. IR (KBr): ν = 1713 (s), 1466 (w), 1381 (m), 1265 (w), 1196 (m), 1119 (m), 1057 (w), 1003 (w), 980 (w), 748 (m) cm^{-1}; ^{1}H NMR (500 MHz, $CDCl_3$): δ = 8.45 (d, J = 7.9, 1H, H-8), 7.82 (d, J = 7.9, 1H, H-5), 7.56-7.39 (m, 3H, H-6 + H-7 + H-5' on furyl ring), 6.52-6.46 (m, 1H, furyl ring), 6.43-6.36 (m, 1H, furyl ring), 5.73 (dd, J = 10.8, 4.0, 1H, OCH), 3.71 (dd, J = 17.2, 10.8, 1H, OCHCH*H*), 3.43 (dist dd, J = 17.2, 4.0, 1H, OCHCH*H*); ^{13}C NMR (125 MHz, $CDCl_3$): δ = 160.2, 152.0, 150.1, 143.3, 137.8, 136.3, 126.1, 125.6, 124.6, 122.1, 121.4, 110.7, 109.4, 73.1, 29.0; GC-MS: m/z = 270 (M^+, 34), 174 (100), 146 (14), 102 (21); HRMS-ESI (m/z): [$(M+H)^+$] calcd for $(C_{15}H_{11}O_3S)^+$: 271.0423; found, 271.0421.

References

1. Ziccarelli, I.; Mancuso, R.; Santandrea, D.; Altomare, A.; Olivieri, D.; Carfagna, C.; Gabriele, B. *J. Catal.* **2023**, *427*, 115101.

2. Liu, J.; Chen, G.; Xing, J.; Liao, J. *Tetrahedron: Asymm.* **2011**, *22,* 575-579.

3. Sherwood, A. M.; Williamson, S.E.; Johnson, S. N.; Ylmaz, A.; Day, V. W.; Prisinzano, T. *J. Org. Chem.* **2018**, *83*, 980-992.

Copies of HRMS spectra

HRMS (ESI-TOF) *m/z*: [(M+H)$^+$] calcd for $(C_{13}H_{17}OS)^+$: 221.0994; found, 221.1004

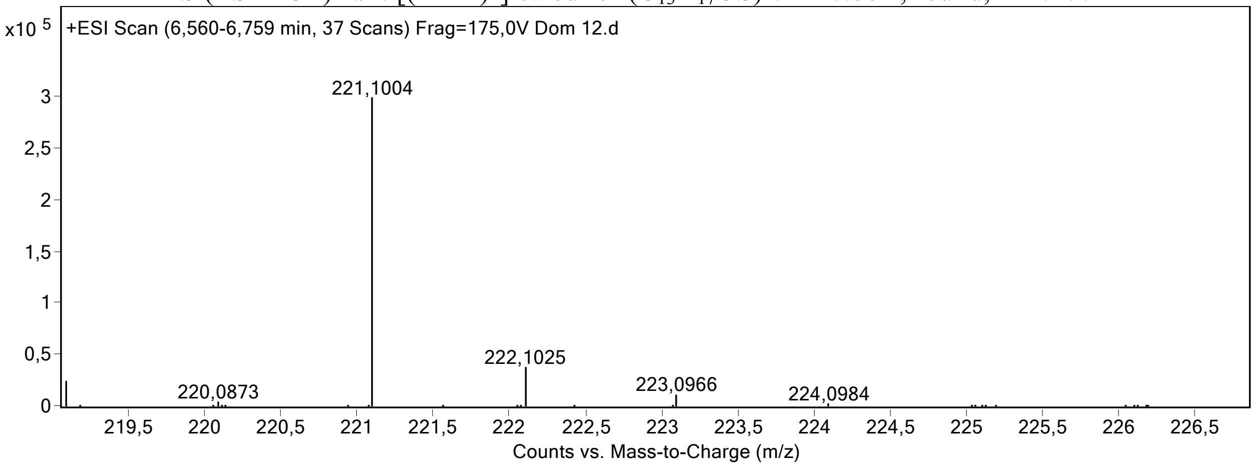

Figure S1. 6-(2-(Methylthio)phenyl)hex-5-yn-3-ol (B_1)

HRMS (ESI-TOF) *m/z*: [(M+H)$^+$] calcd for $(C_{11}H_{12}FOS)^+$: 211.0587; found, 211.0595

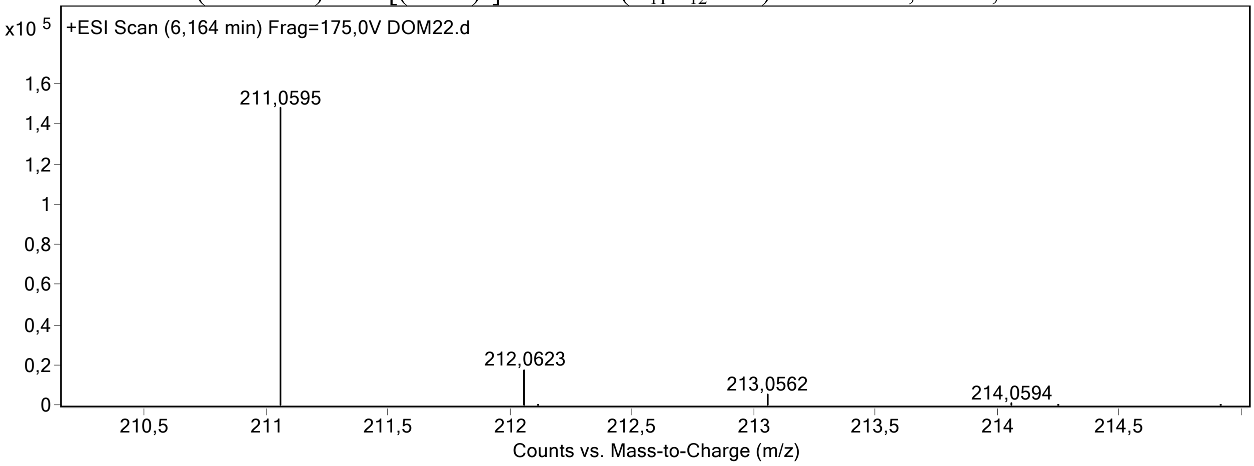

Figure S2. 4-(4-Fluoro-2-(methylthio)phenyl)but-3-yn-1-ol (B_2)

HRMS (ESI-TOF) *m/z*: [(M-H_2O+H)$^+$] calcd for $(C_{18}H_{17}S)^+$: 265.1045; found, 265.1053

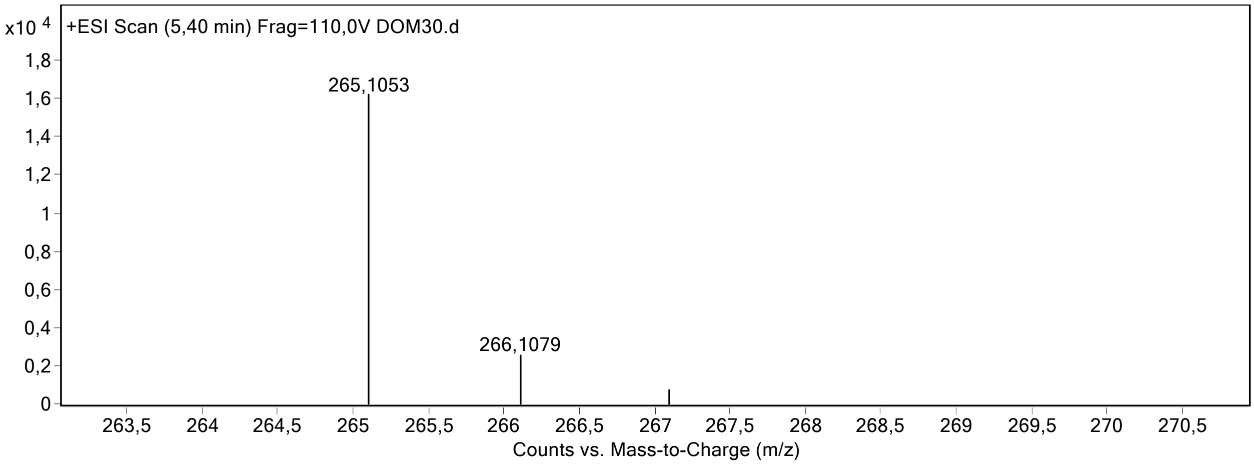

Figure S3. 5-(2-(Methylthio)phenyl)-2-phenylpent-4-yn-2-ol (B_3)

HRMS (ESI-TOF) *m/z*: [(M-H_2O+H)$^+$] calcd for ($C_{17}H_{15}S$)$^+$: 251.0889; found, 251.0880

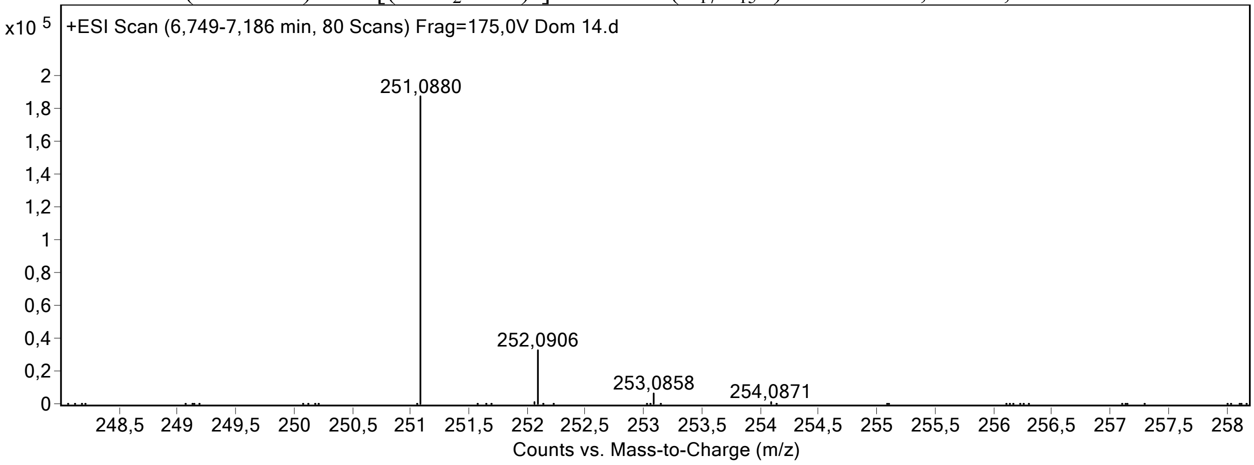

Figure S4. 4-(2-(Methylthio)phenyl)-1-phenylbut-3-yn-1-ol (B_4)

HRMS (ESI-TOF) *m/z*: [(M-H_2O+H)$^+$] calcd for ($C_{15}H_{13}OS$)$^+$: 241.0681; found, 241.0690

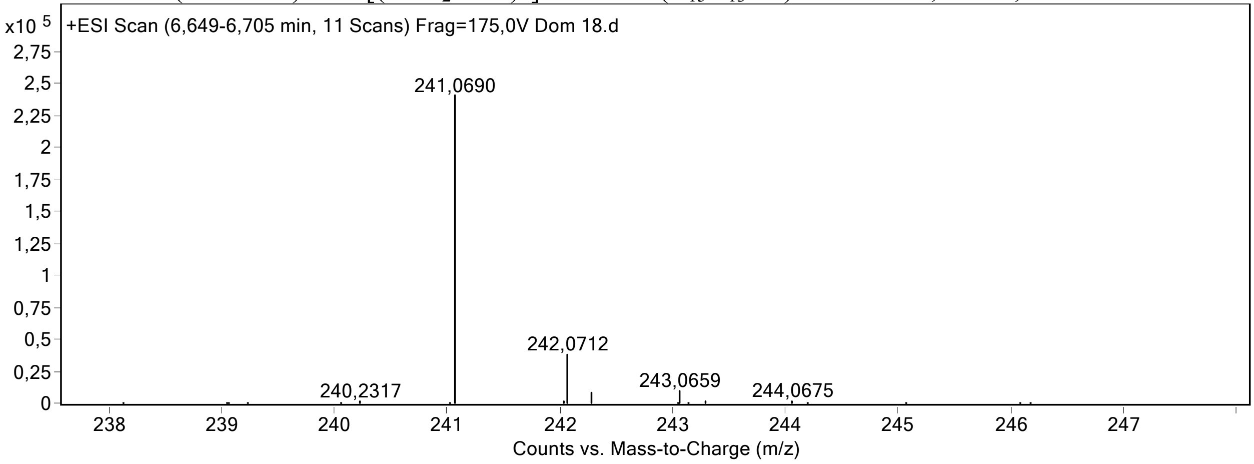

Figure S5. 1-(Furan-2-yl)-4-(2-(methylthio)phenyl)but-3-yn-1-ol (B_5)

HRMS (ESI-TOF) *m/z*: [(M+H)$^+$] calcd for ($C_{13}H_{13}O_2S$)$^+$: 233.0630; found, 233.0615

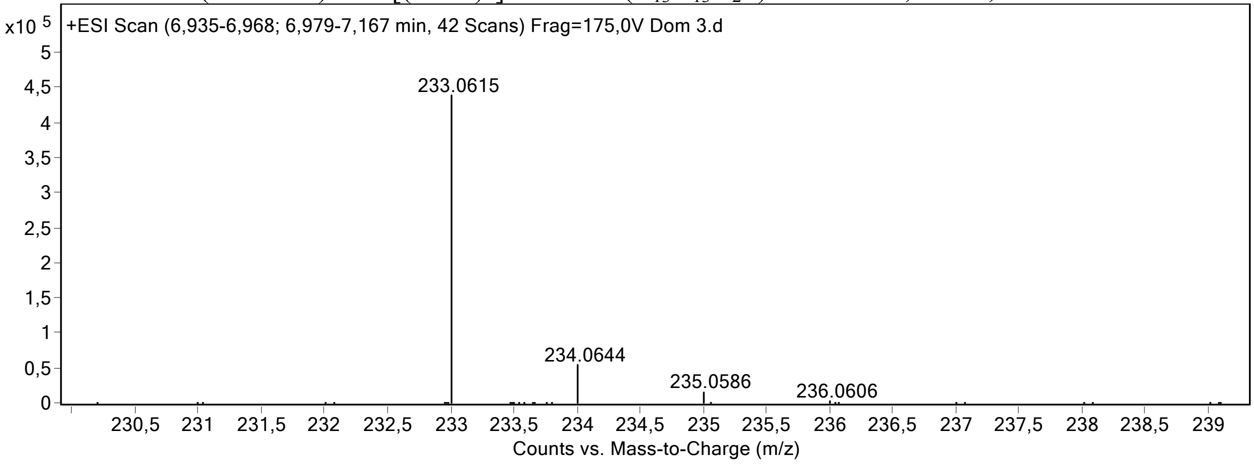

Figure S6. 3-Ethyl-3,4-dihydro-1*H*-benzo[4,5]thieno[3,2-*c*]pyran-1-one (A_1)

HRMS (ESI-TOF) *m/z*: [(M+H)$^+$] calcd for ($C_{11}H_8FO_2S$)$^+$: 223.0224; found, 223.0234

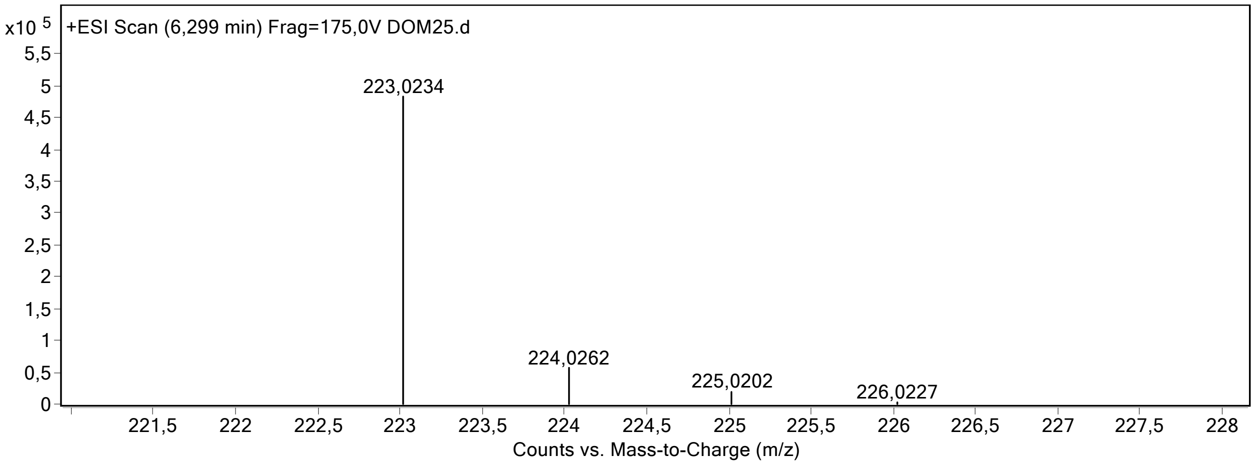

Figure S7. 7-Fluoro-3,4-dihydro-1*H*-benzo[4,5]thieno[3,2-*c*]pyran-1-one (A_2)

HRMS (ESI-TOF) *m/z*: [(M+H)$^+$] calcd for $(C_{18}H_{15}O_2S)^+$: 295.0787; found, 295.0797

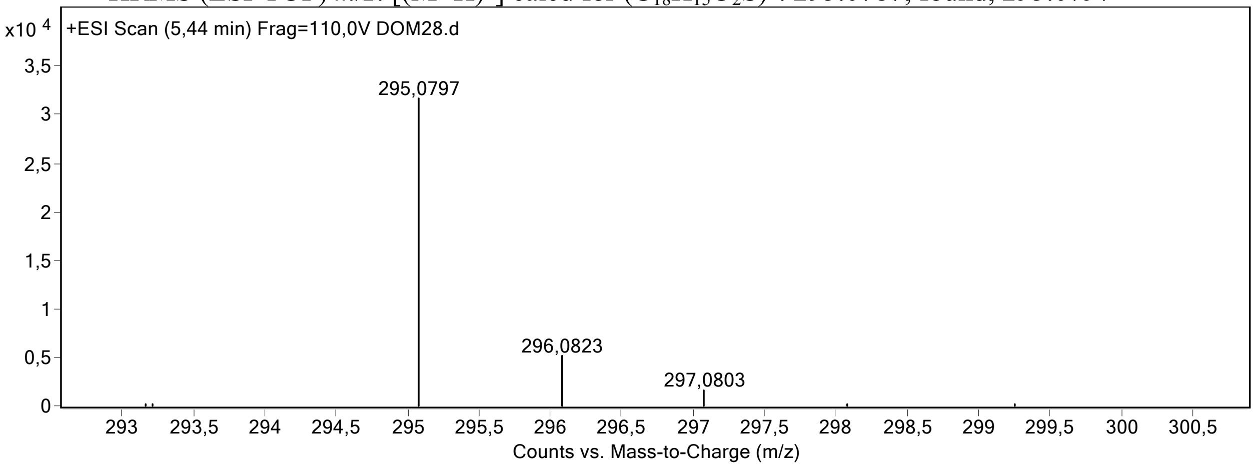

Figure S8. 3-Methyl-3-phenyl-3,4-dihydro-1*H*-benzo[4,5]thieno[3,2-*c*]pyran-1-one (A_3)

HRMS (ESI-TOF) *m/z*: [(M+H)+] calcd for $(C_{17}H_{13}O_2S)^+$: 281.0630; found, 281.0647

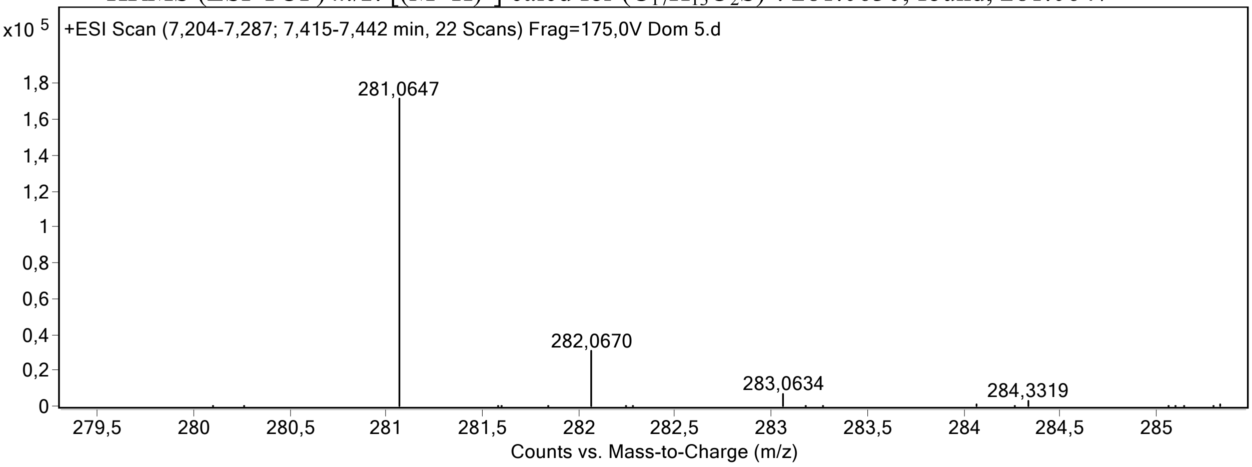

Figure S9. 3-Phenyl-3,4-dihydro-1*H*-benzo[4,5]thieno[3,2-*c*]pyran-1-one (A_4)

HRMS (ESI-TOF) *m/z*: [(M+H)+] calcd for $(C_{15}H_{11}O_3S)^+$: 271.0423; found, 271.0421

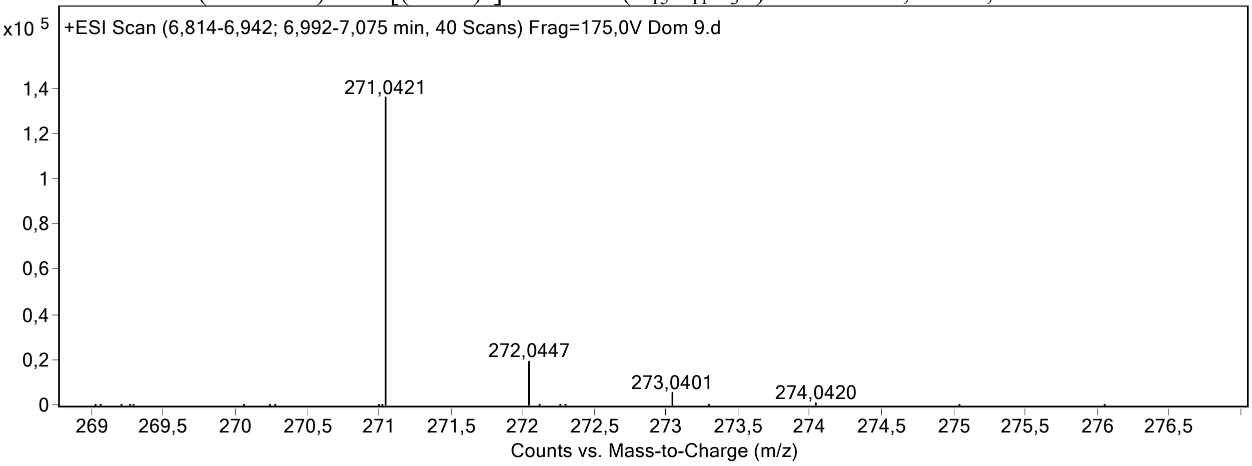

Figure S10. 3-(Furan-2-yl)-3,4-dihydro-1*H*-benzo[4,5]thieno[3,2-*c*]pyran-1-one (A_5)

Copies of ^{1}H NMR and ^{13}CNMR Spectra

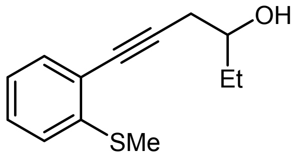

^{1}H NMR ($CDCl_3$, 500 MHz)

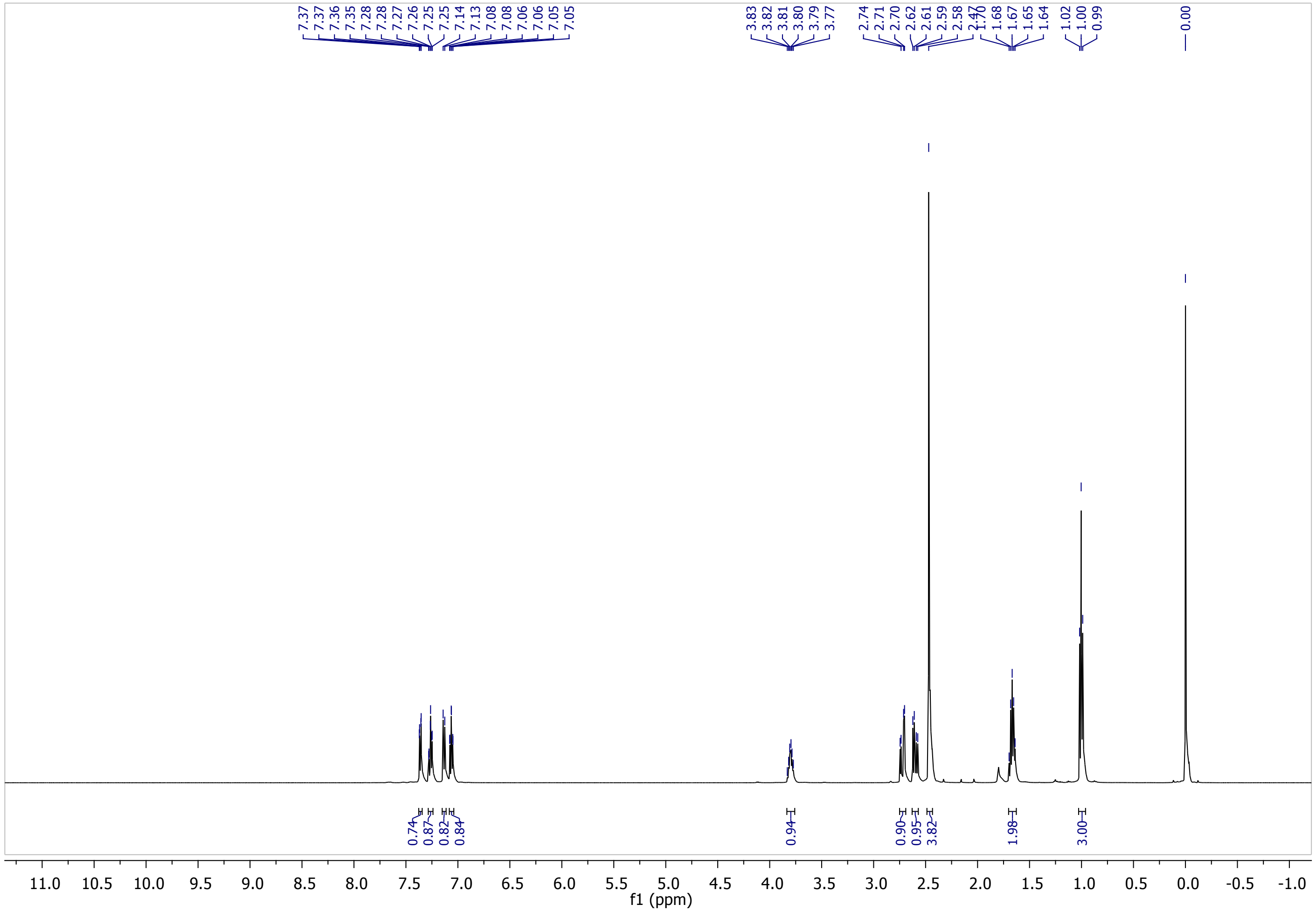

Figure S11. 6-(2-(Methylthio)phenyl)hex-5-yn-3-ol (**B$_1$**)

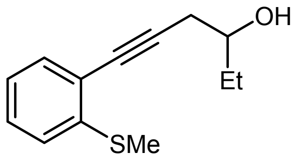

^{13}C NMR ($CDCl_3$, 125 MHz)

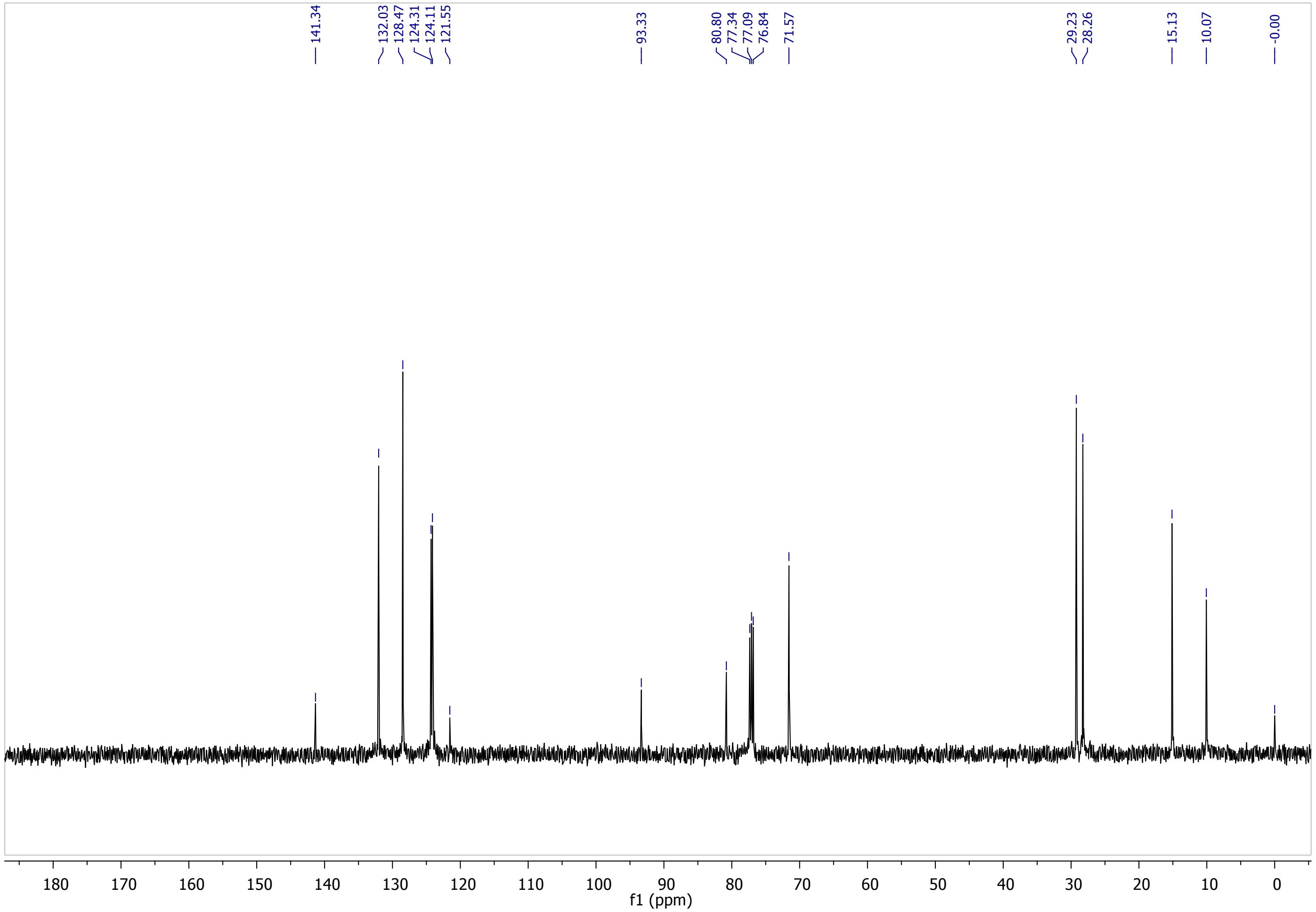

Figure S12. 6-(2-(Methylthio)phenyl)hex-5-yn-3-ol (**B$_1$**)

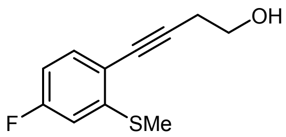

^{1}H NMR ($CDCl_3$, 500 MHz)

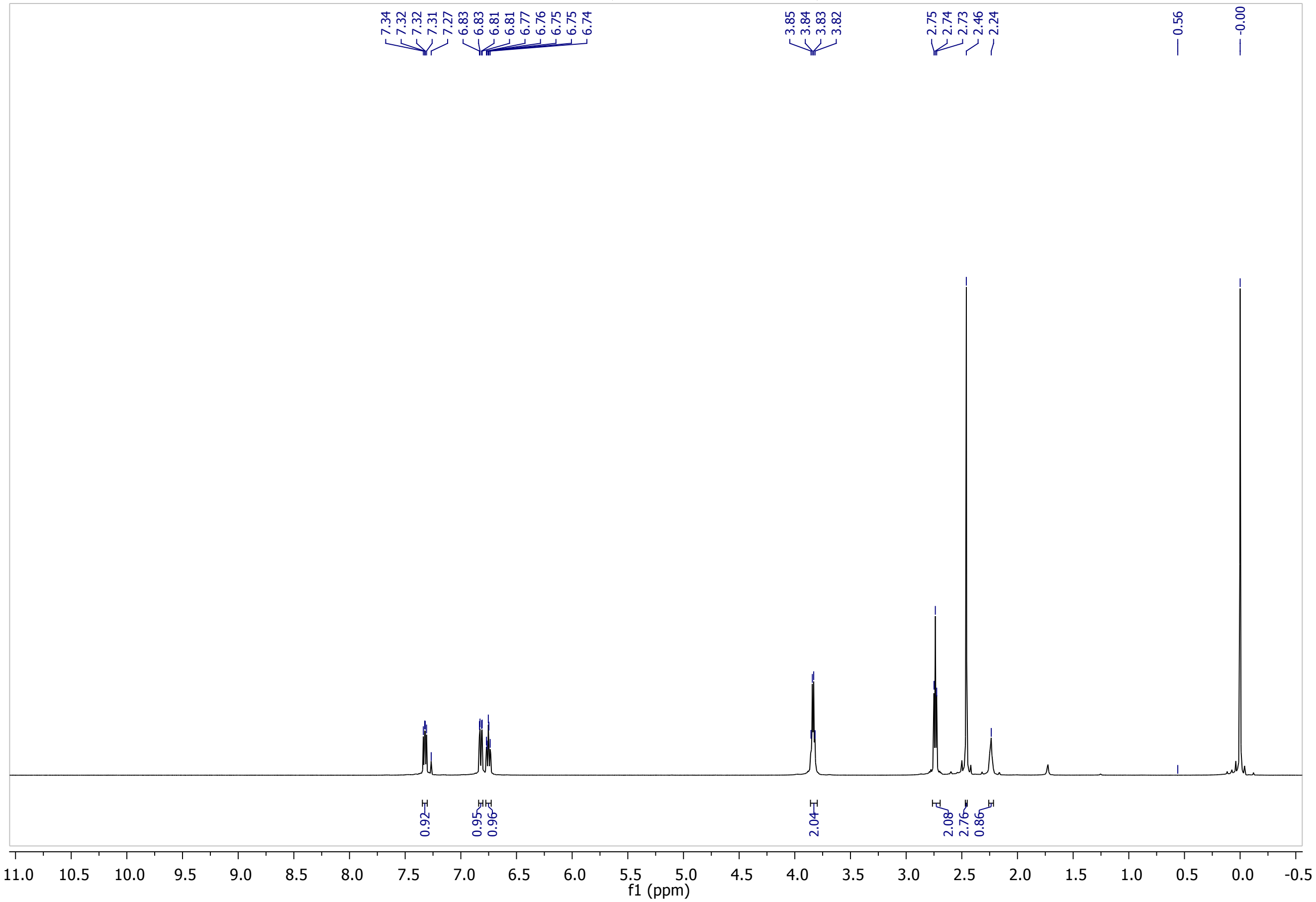

Figure S 13. 4-(4-Fluoro-2-(methylthio)phenyl)but-3-yn-1-ol (**B$_2$**)

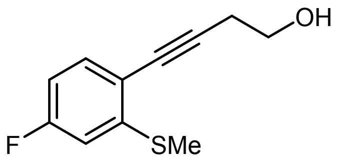

^{13}C NMR ($CDCl_3$, 125 MHz)

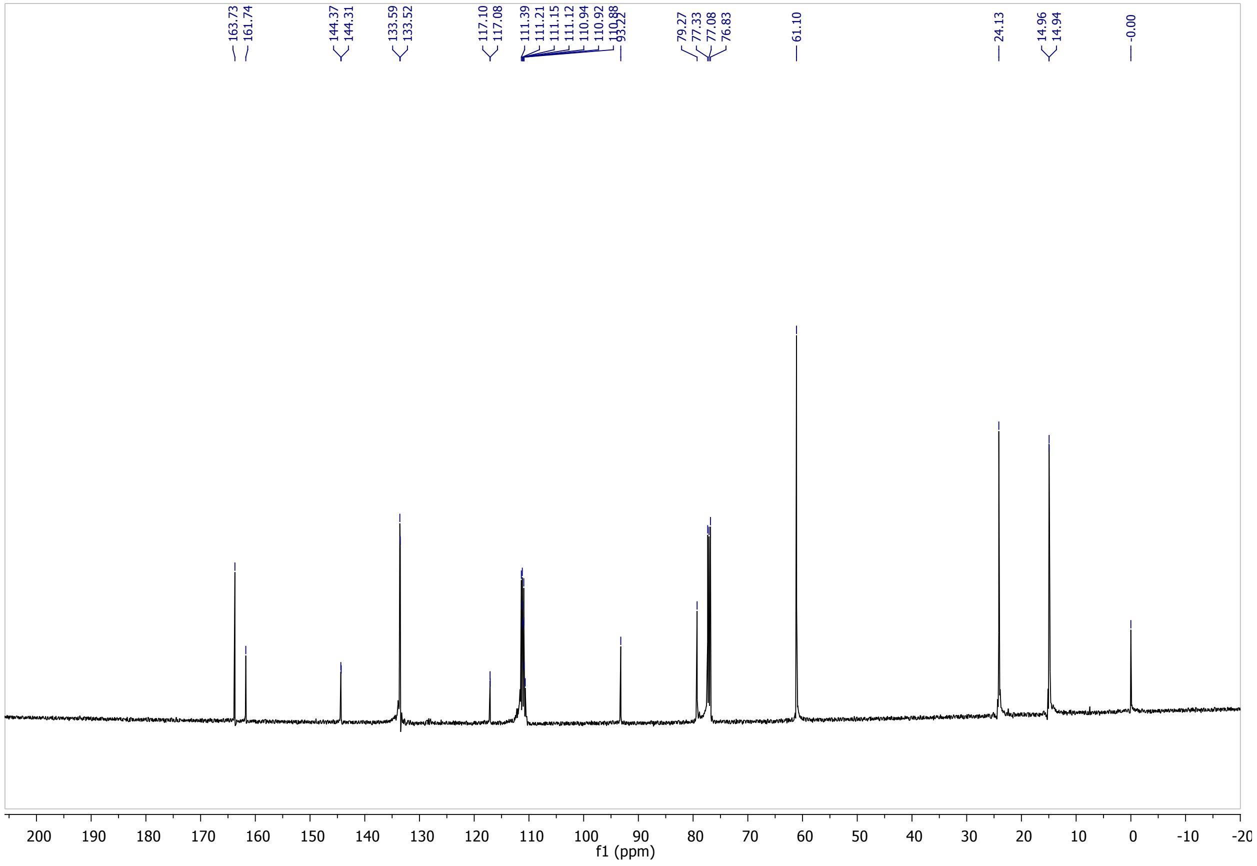

Figure S 14. 4-(4-Fluoro-2-(methylthio)phenyl)but-3-yn-1-ol (**B$_2$**)

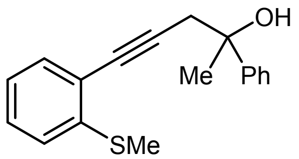

^{1}H NMR ($CDCl_3$, 500 MHz)

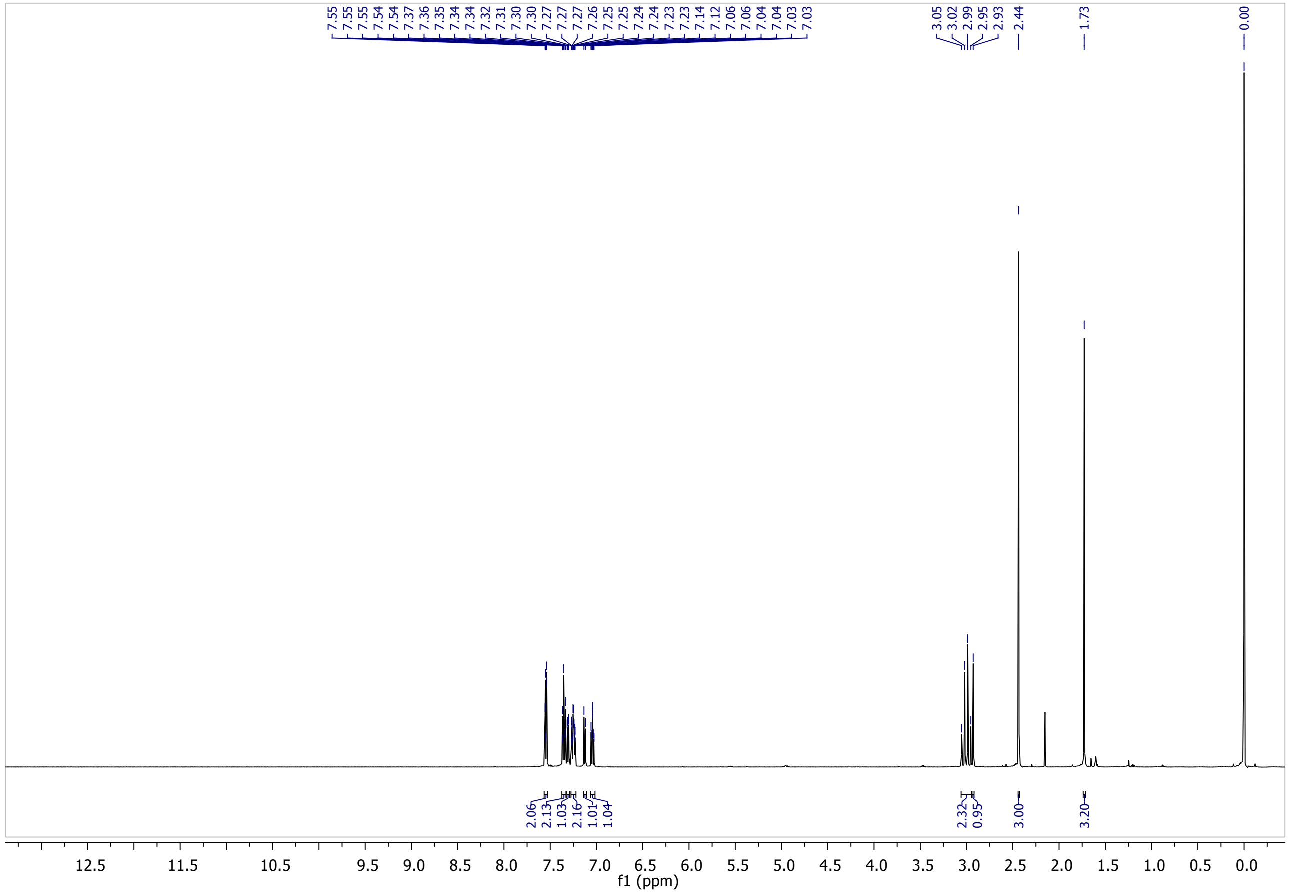

Figure S 15. 5-(2-(Methylthio)phenyl)-2-phenylpent-4-yn-2-ol (**B$_3$**)

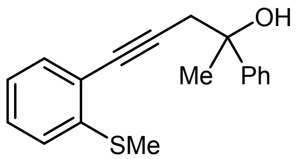

^{13}C NMR ($CDCl_3$, 125 MHz)

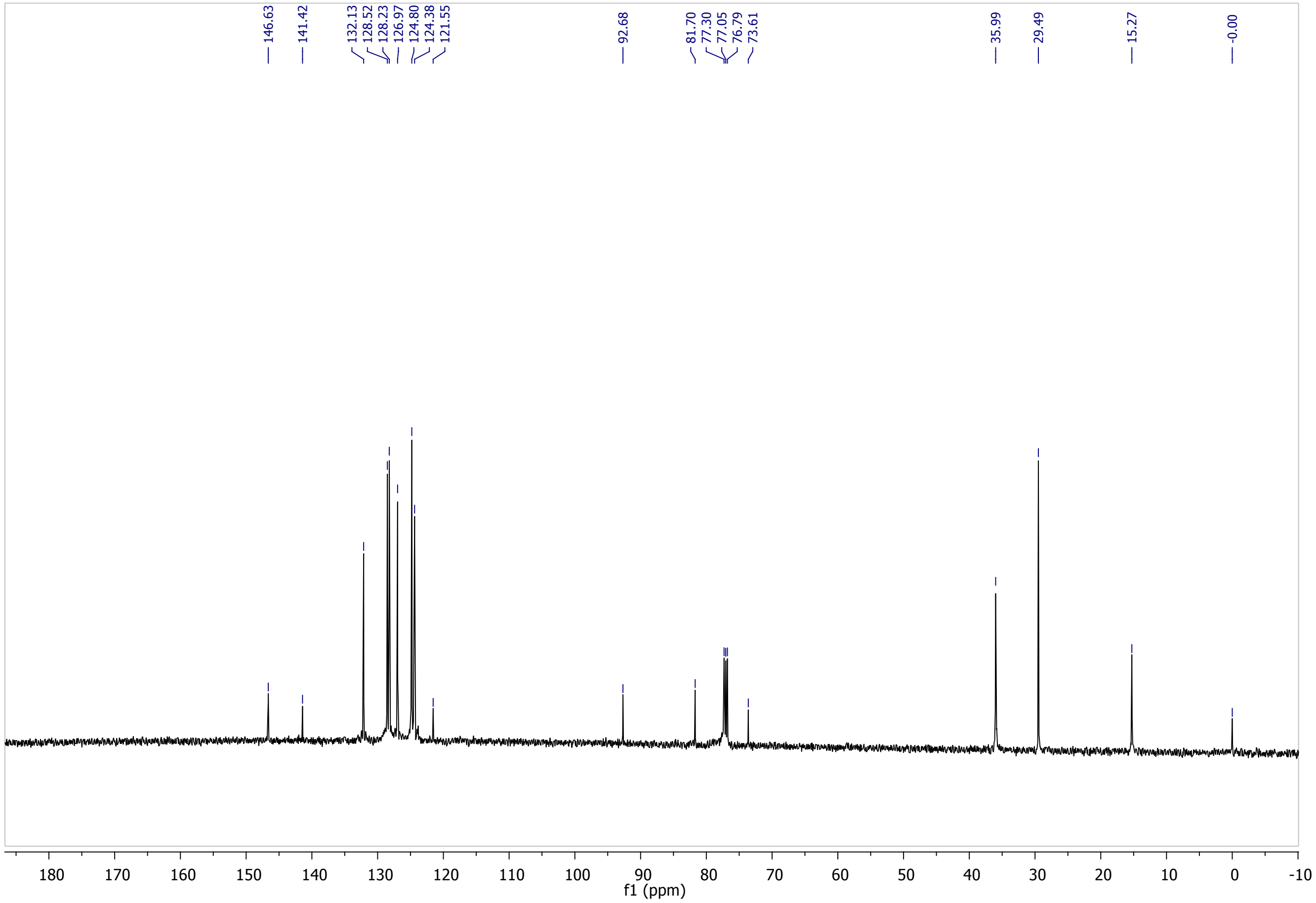

Figure S 16. 5-(2-(Methylthio)phenyl)-2-phenylpent-4-yn-2-ol (**B$_3$**)

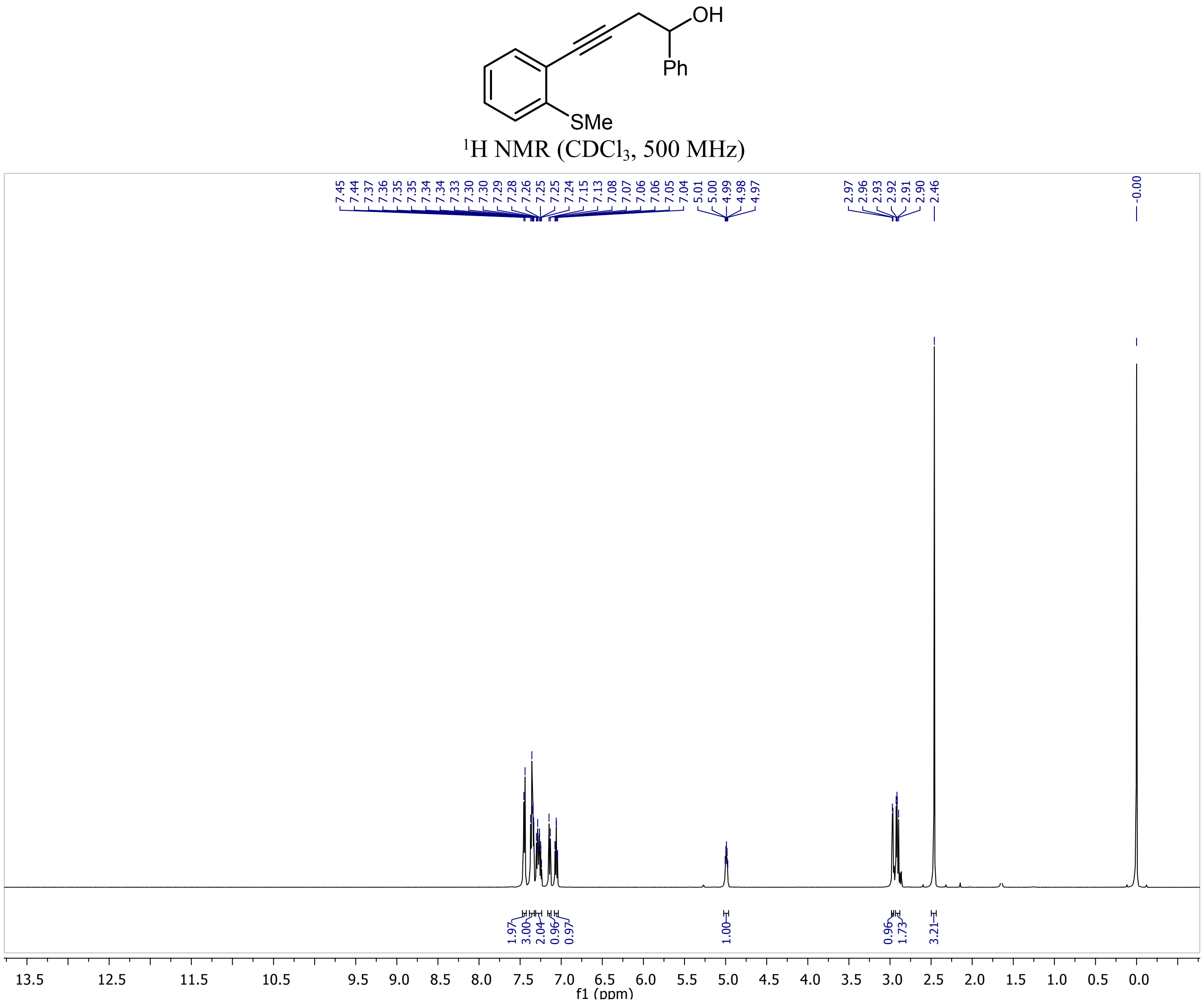

Figure S 17. 4-(2-(Methylthio)phenyl)-1-phenylbut-3-yn-1-ol (**B_4**)

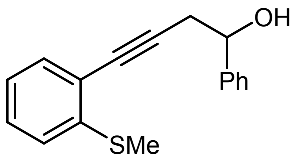

^{13}C NMR ($CDCl_3$, 125 MHz)

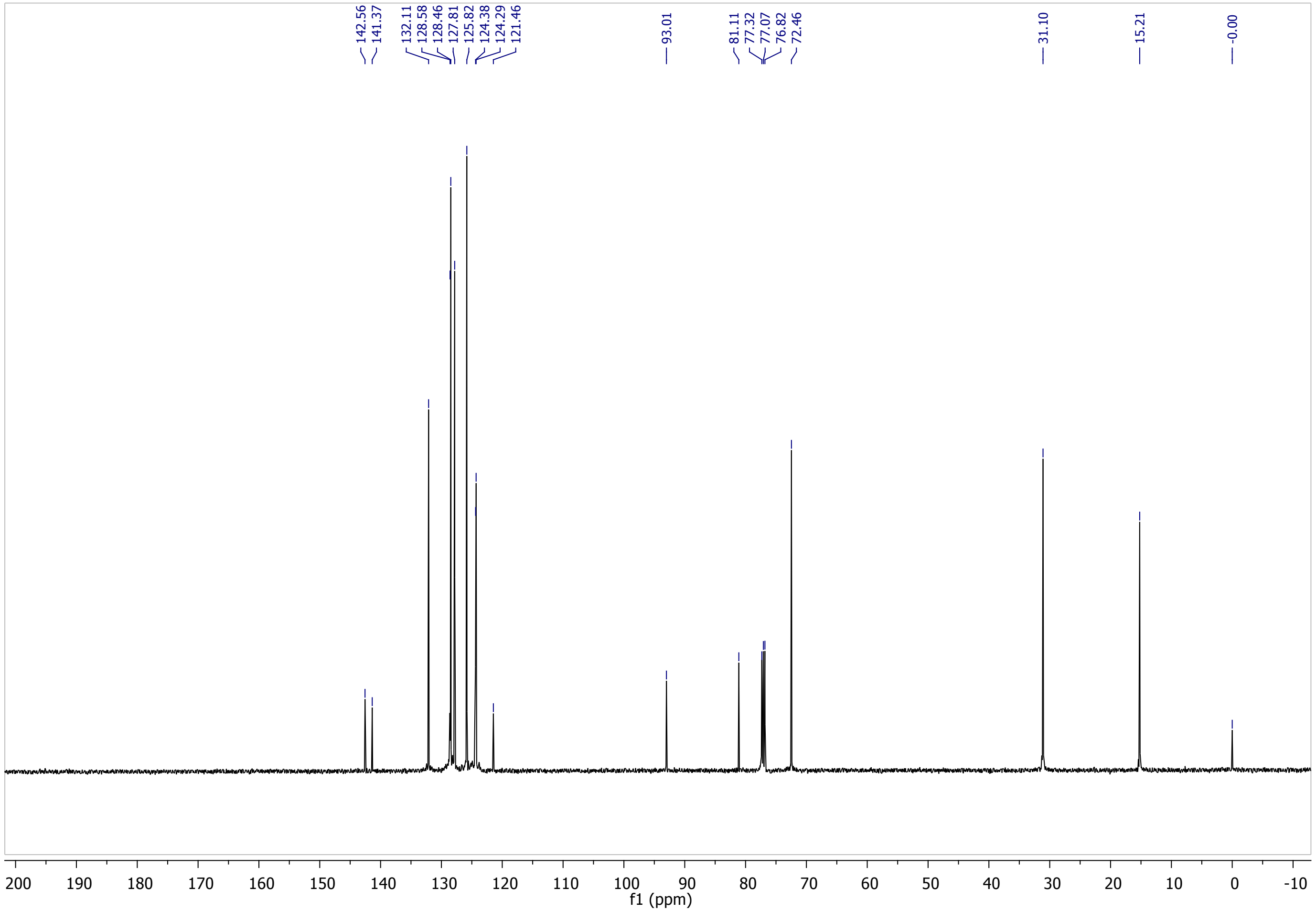

Figure S 18. 4-(2-(Methylthio)phenyl)-1-phenylbut-3-yn-1-ol (**B$_4$**)

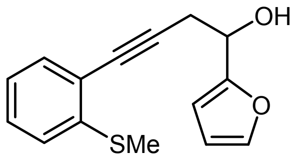

^{1}H NMR ($CDCl_3$, 500 MHz)

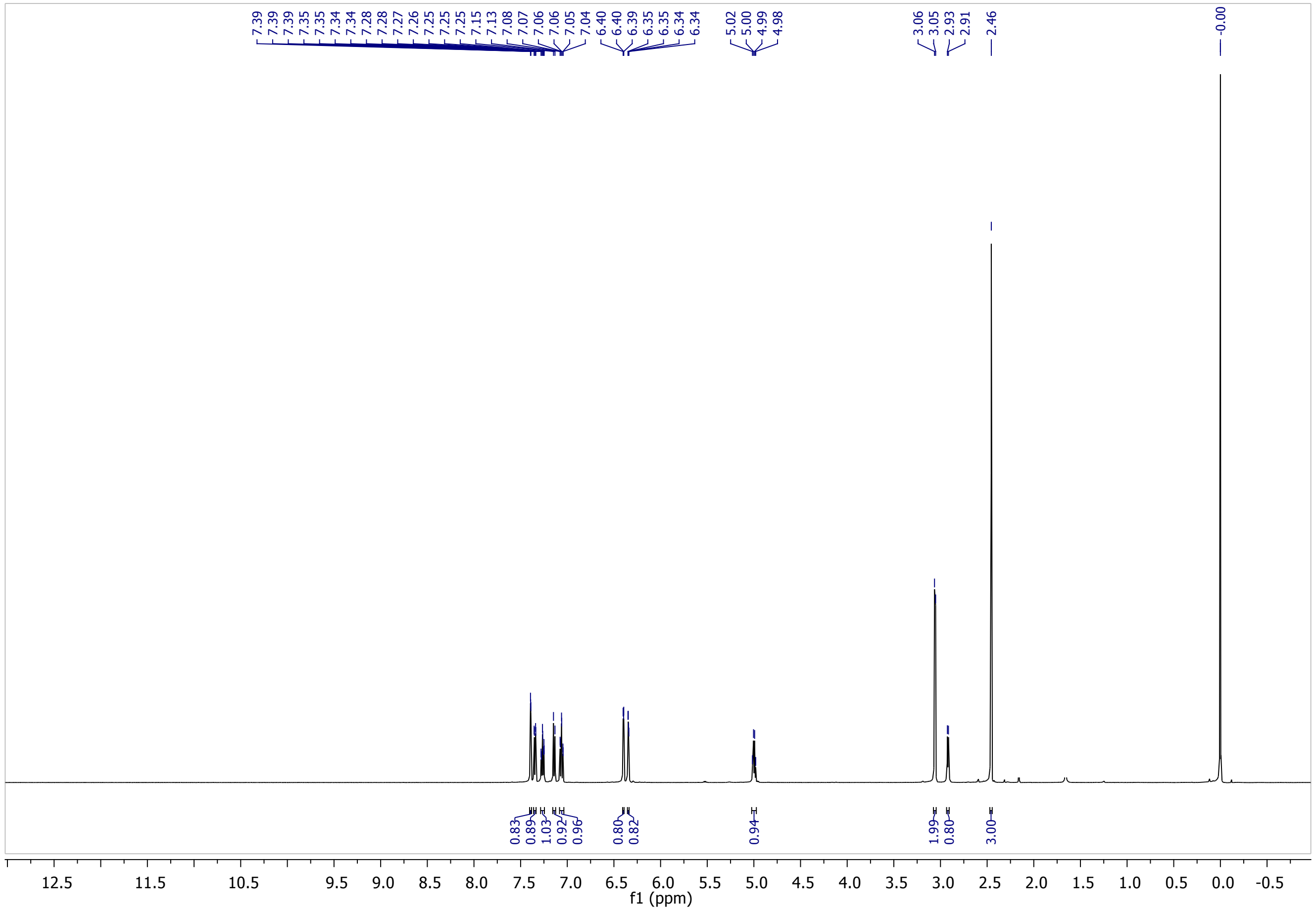

Figure S 19. 1-(Furan-2-yl)-4-(2-(methylthio)phenyl)but-3-yn-1-ol (**B$_5$**)

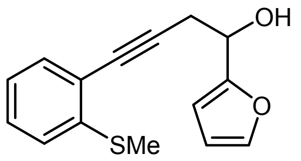

^{13}C NMR ($CDCl_3$, 125 MHz)

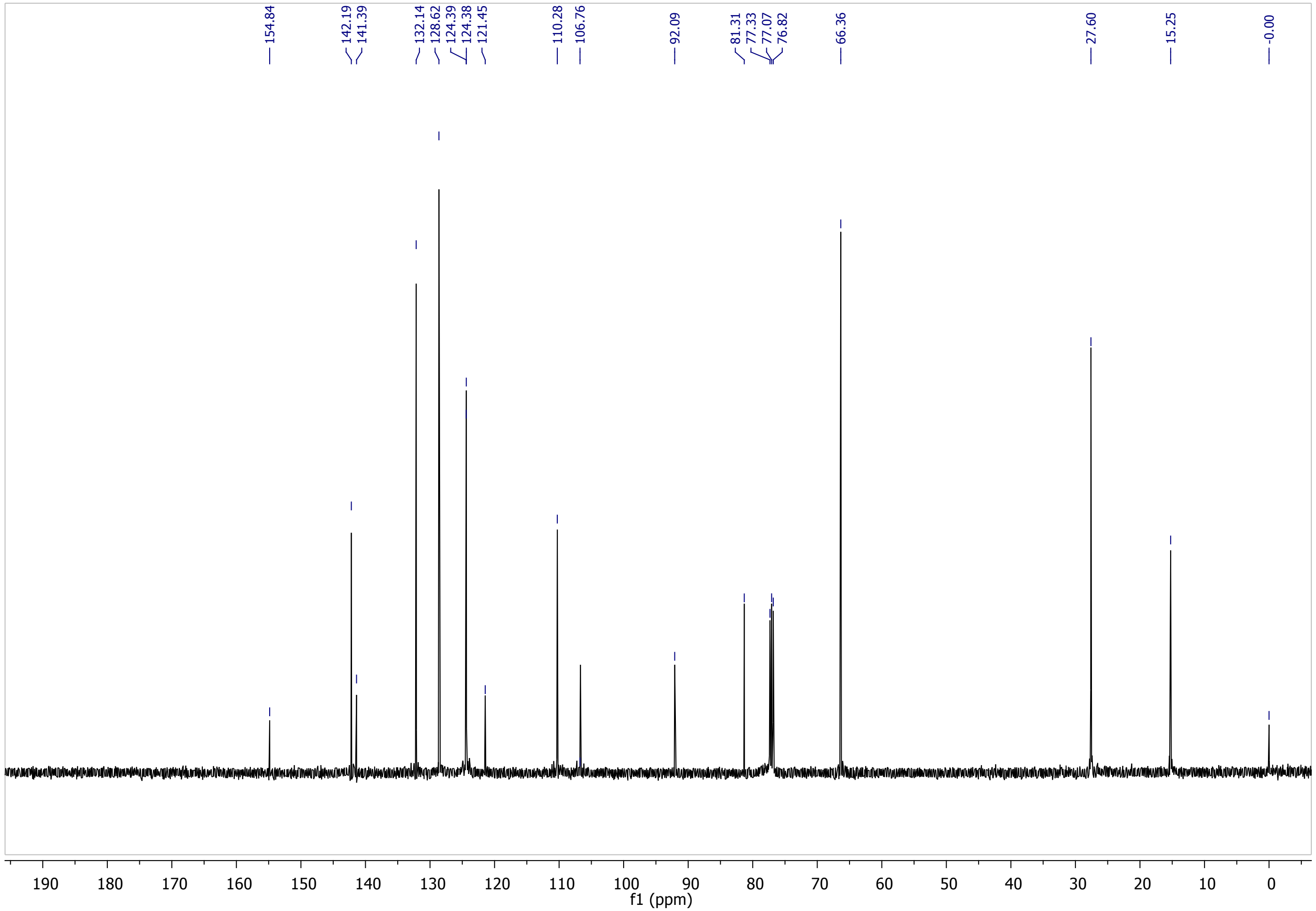

Figure S 20. 1-(Furan-2-yl)-4-(2-(methylthio)phenyl)but-3-yn-1-ol (**B$_5$**)

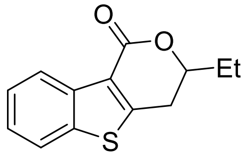

^{1}H NMR ($CDCl_3$, 500 MHz)

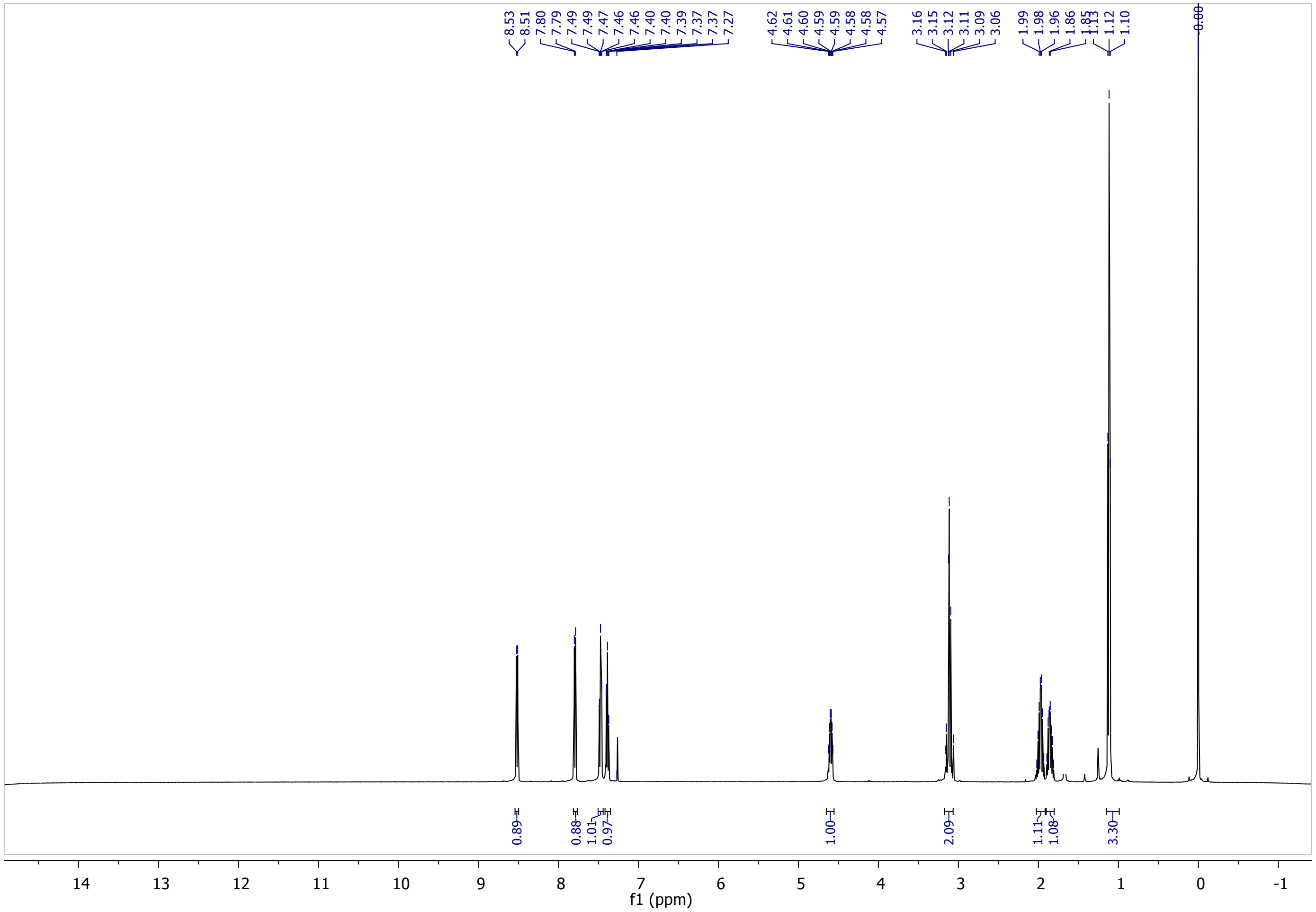

Figure S 21. 3-Ethyl-3,4-dihydro-1*H*-benzo[4,5]thieno[3,2-*c*]pyran-1-one (**A$_1$**)

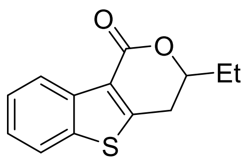

^{13}C NMR ($CDCl_3$, 125 MHz)

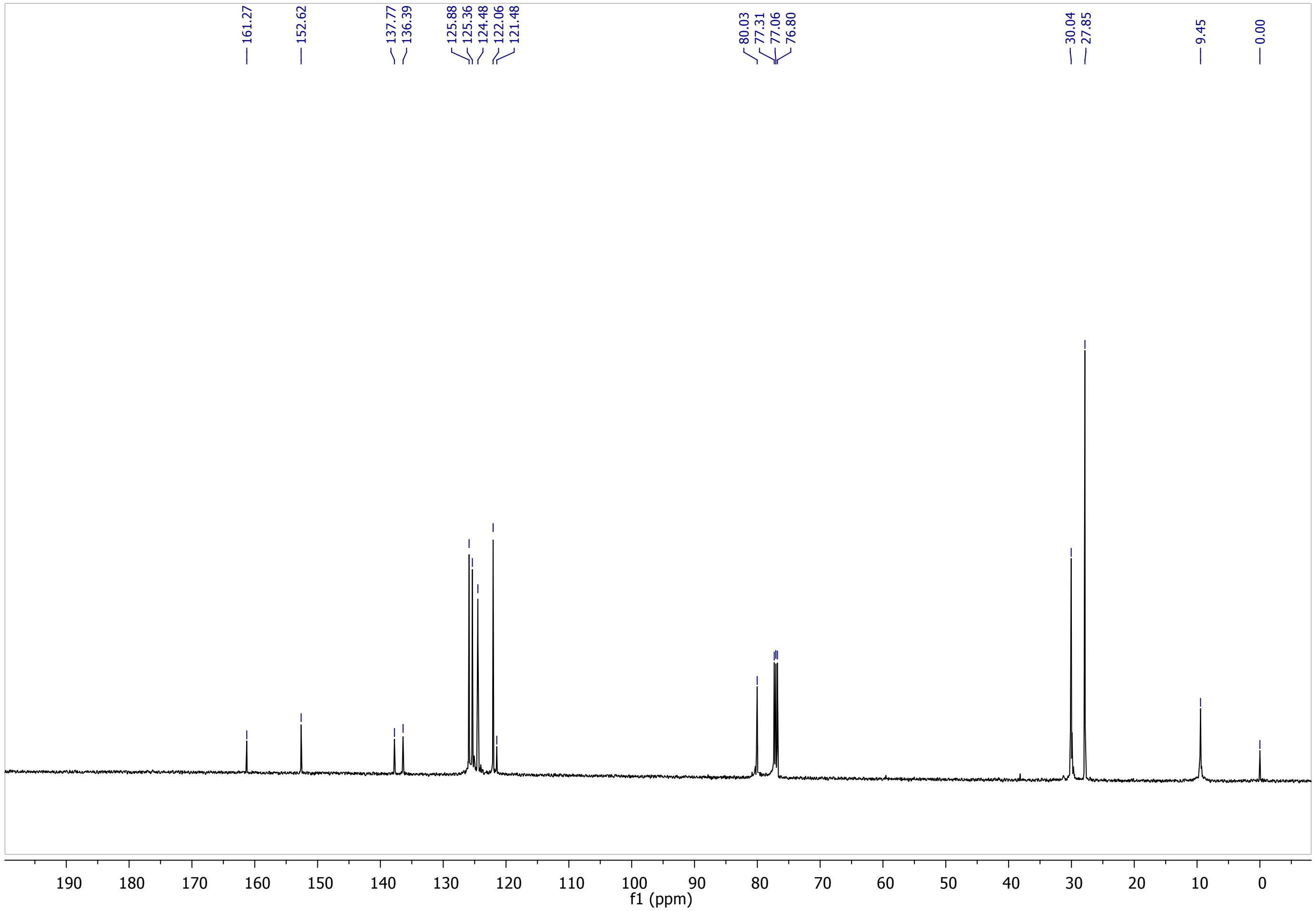

Figure S 22. 3-Ethyl-3,4-dihydro-1*H*-benzo[4,5]thieno[3,2-*c*]pyran-1-one (**A₁**)

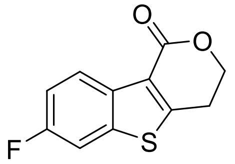

^{1}H NMR ($CDCl_3$, 500 MHz)

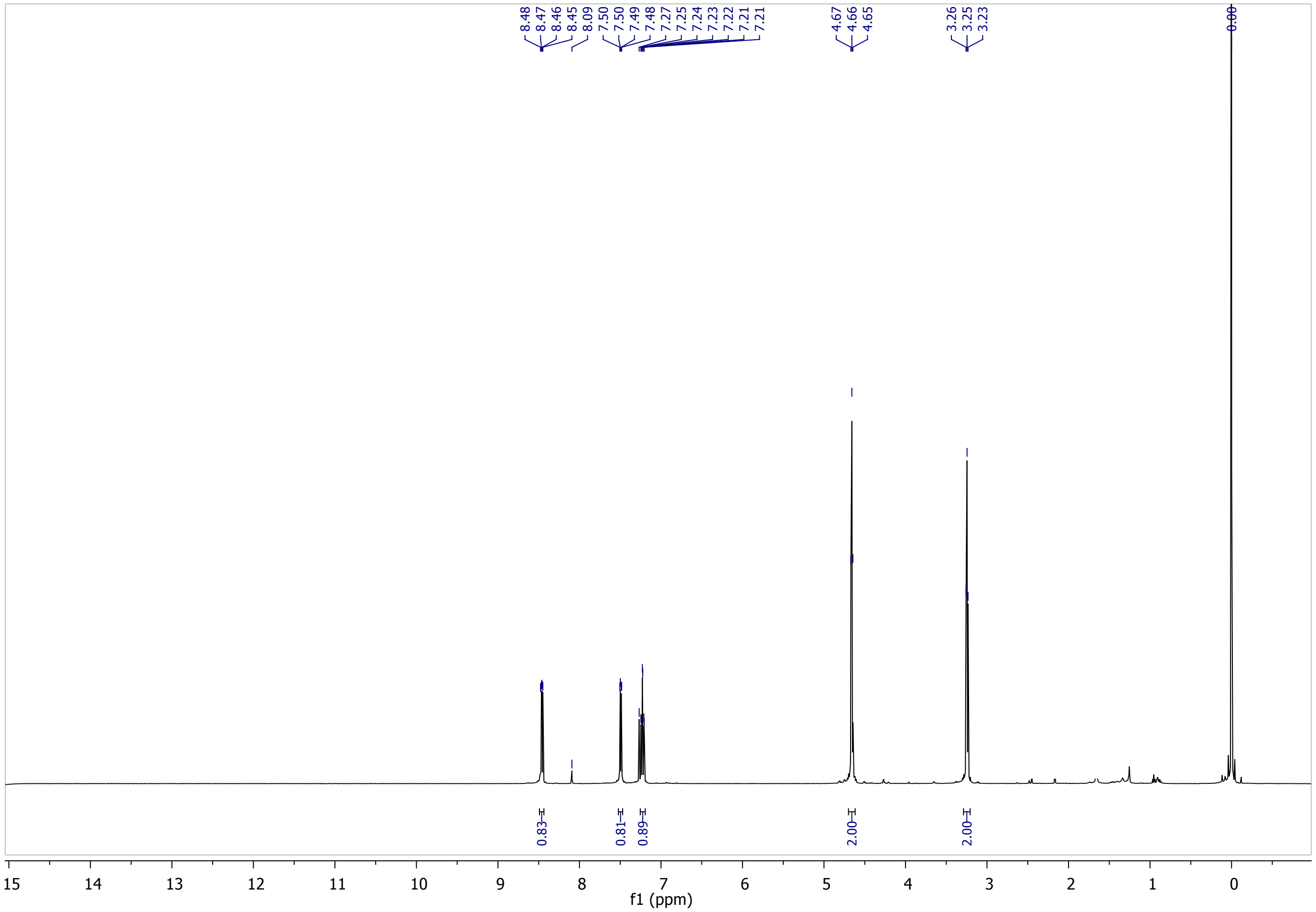

Figure S 23. 7-Fluoro-3,4-Dihydro-1*H*-benzo[4,5]thieno[3,2-*c*]pyran-1-one (**A$_2$**)

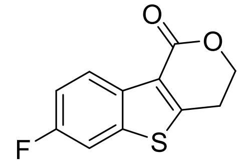

^{13}C NMR ($CDCl_3$, 125 MHz)

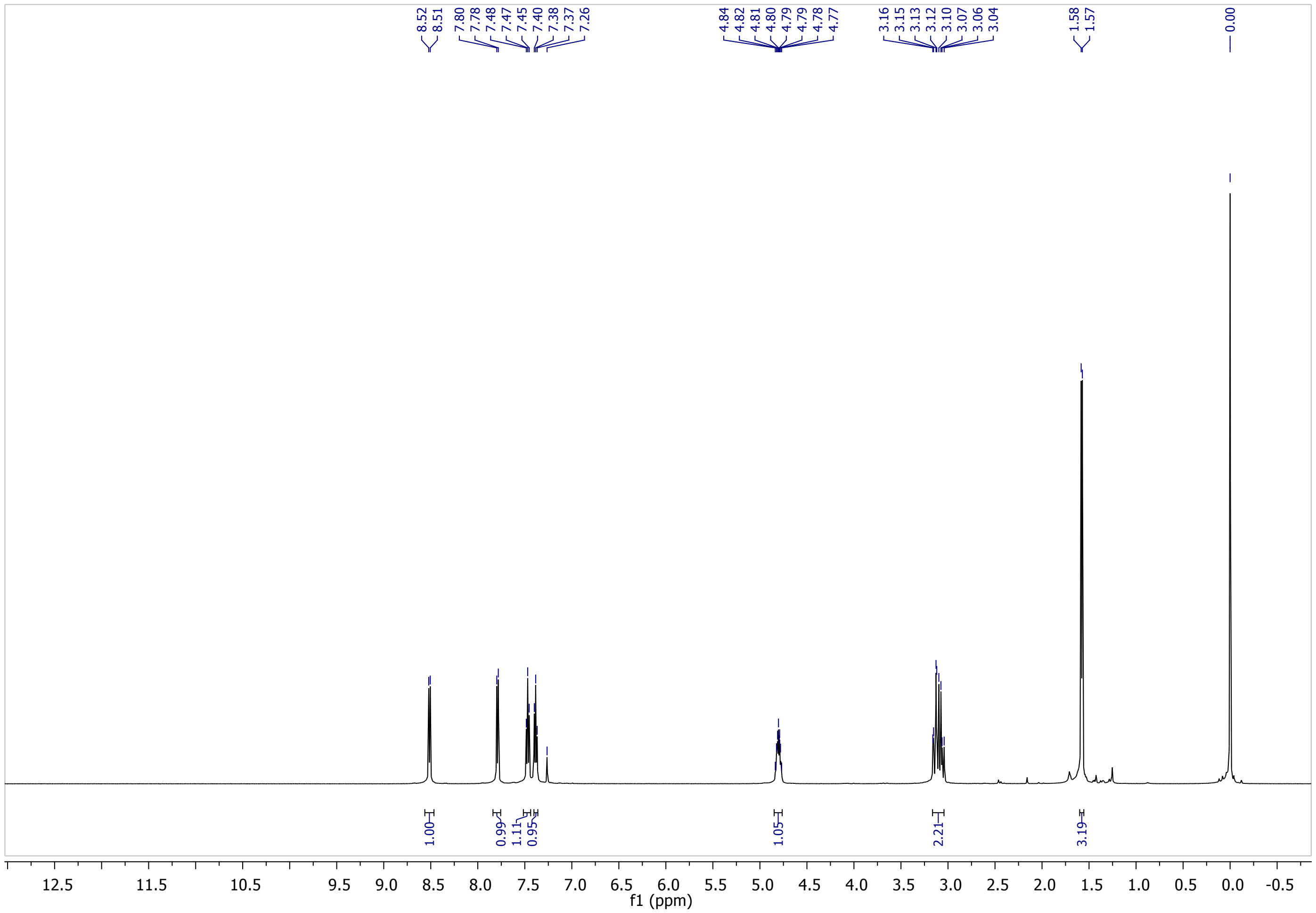

Figure S 24. 7-Fluoro-3,4-Dihydro-1*H*-benzo[4,5]thieno[3,2-*c*]pyran-1-one (**A$_2$**)

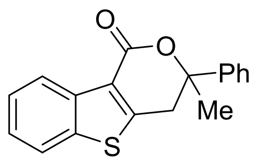

^{1}H NMR ($CDCl_3$, 500 MHz)

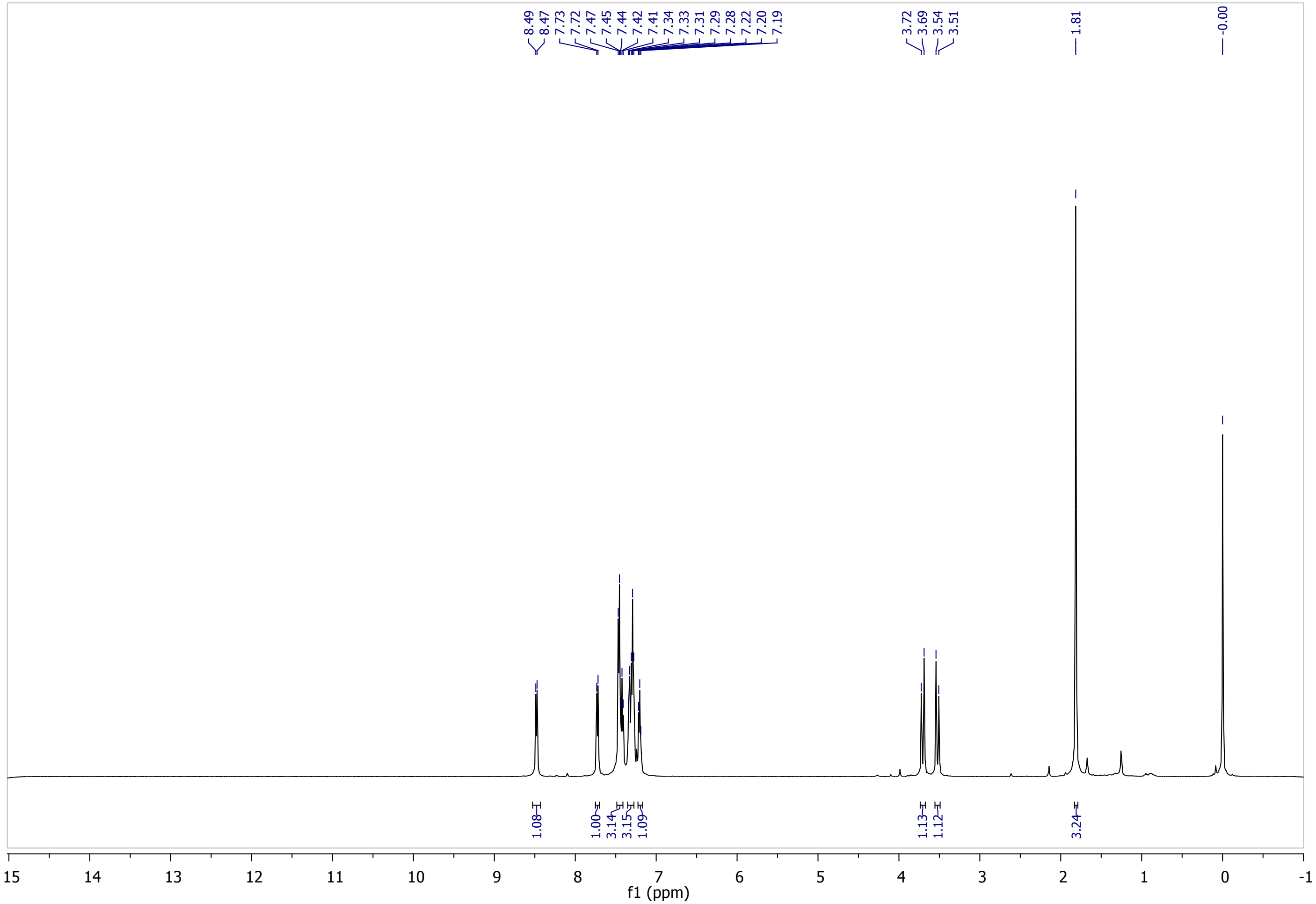

Figure S 25. 3-Methyl-3-phenyl-3,4-dihydro-1*H*-benzo[4,5]thieno[3,2-*c*]pyran-1-one (**A$_3$**)

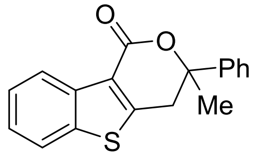

^{13}C NMR ($CDCl_3$, 125 MHz)

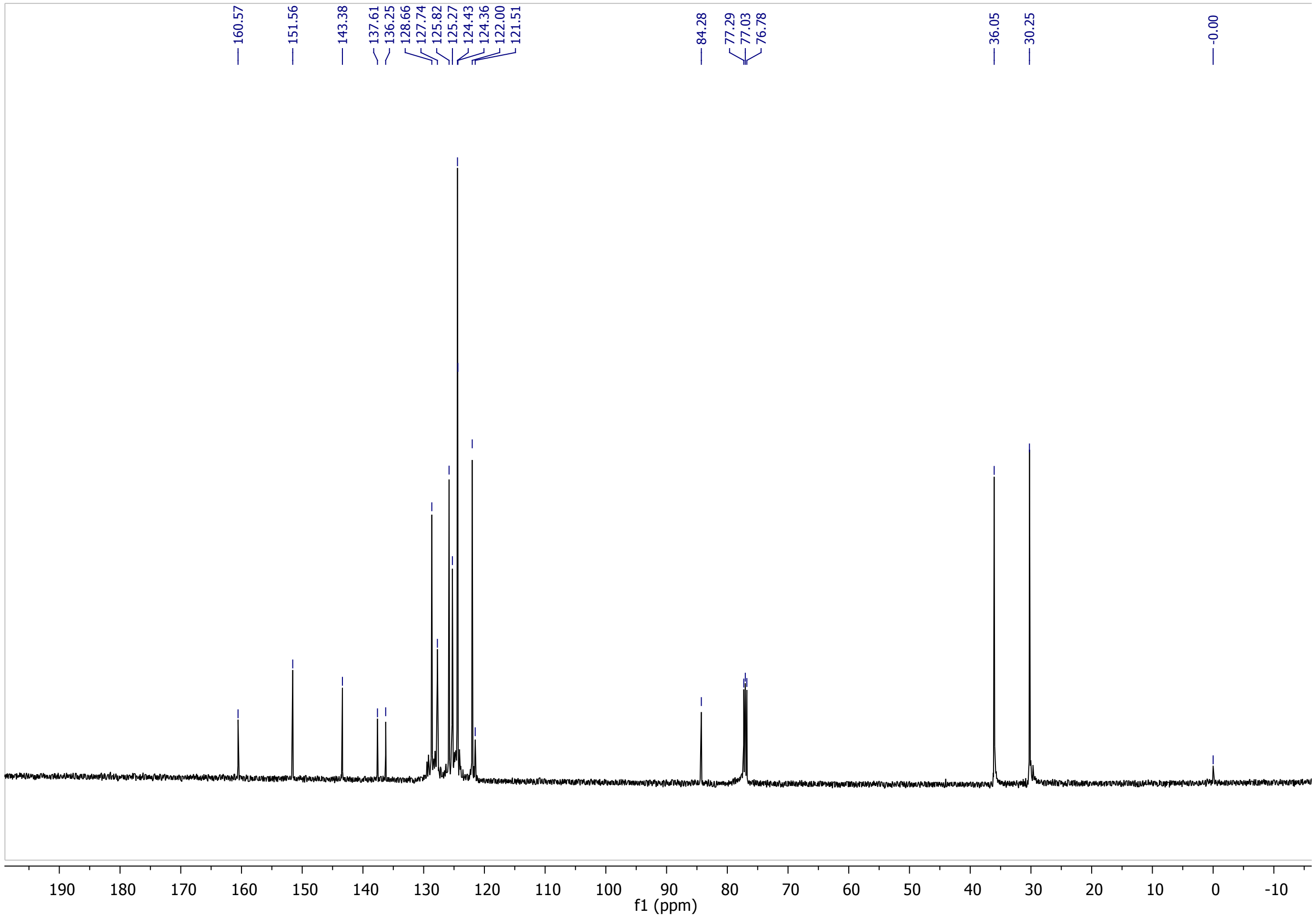

Figure S 26. 3-Methyl-3-phenyl-3,4-dihydro-1*H*-benzo[4,5]thieno[3,2-*c*]pyran-1-one (**A$_3$**)

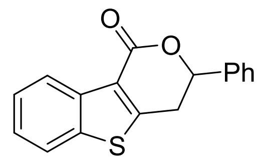

^{1}H NMR ($CDCl_3$, 500 MHz)

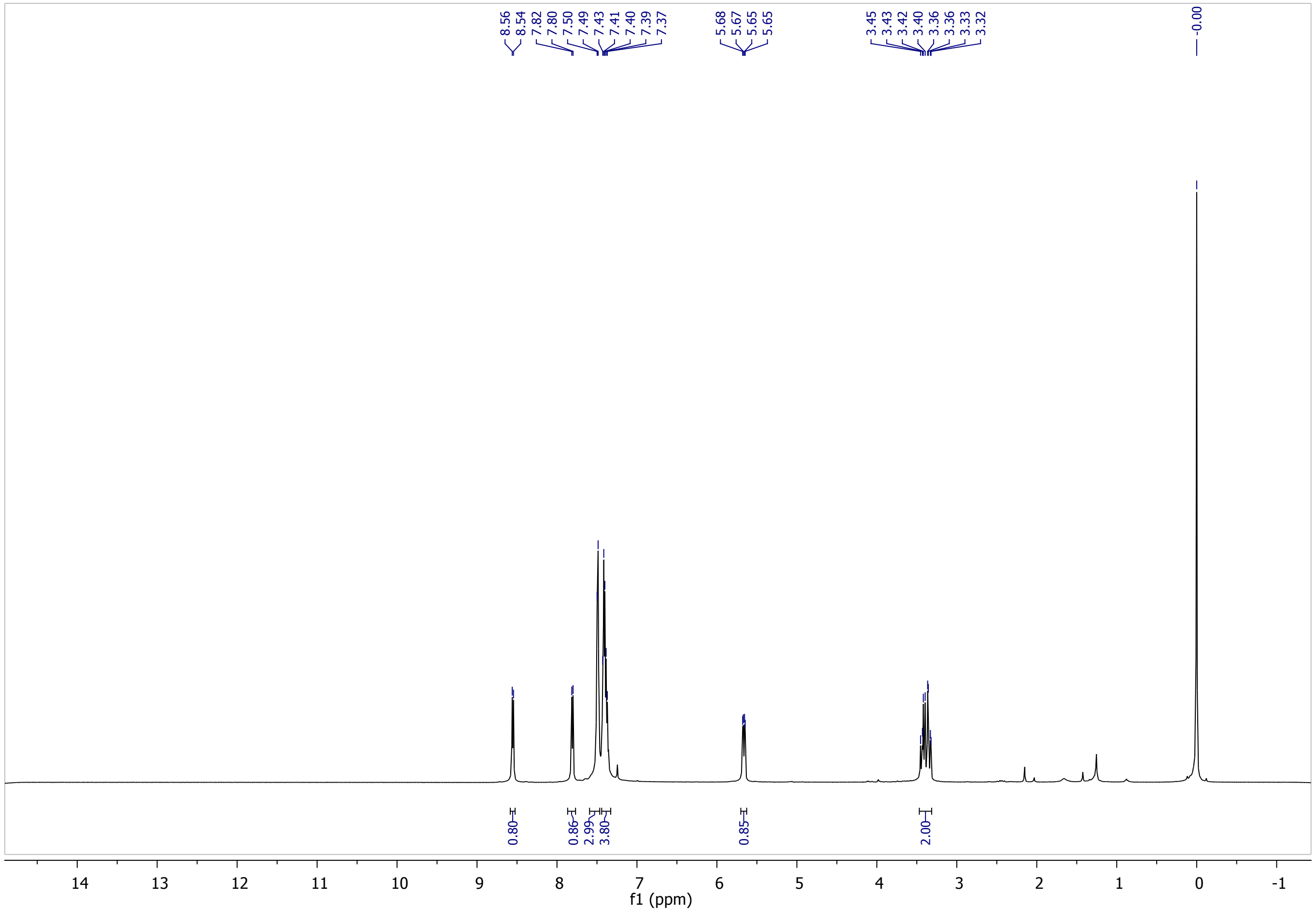

Figure S 27. 3-Phenyl-3,4-dihydro-1*H*-benzo[4,5]thieno[3,2-*c*]pyran-1-one (**A_4**)

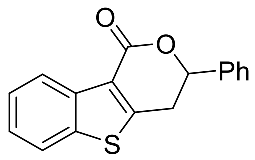

^{13}C NMR ($CDCl_3$, 125 MHz)

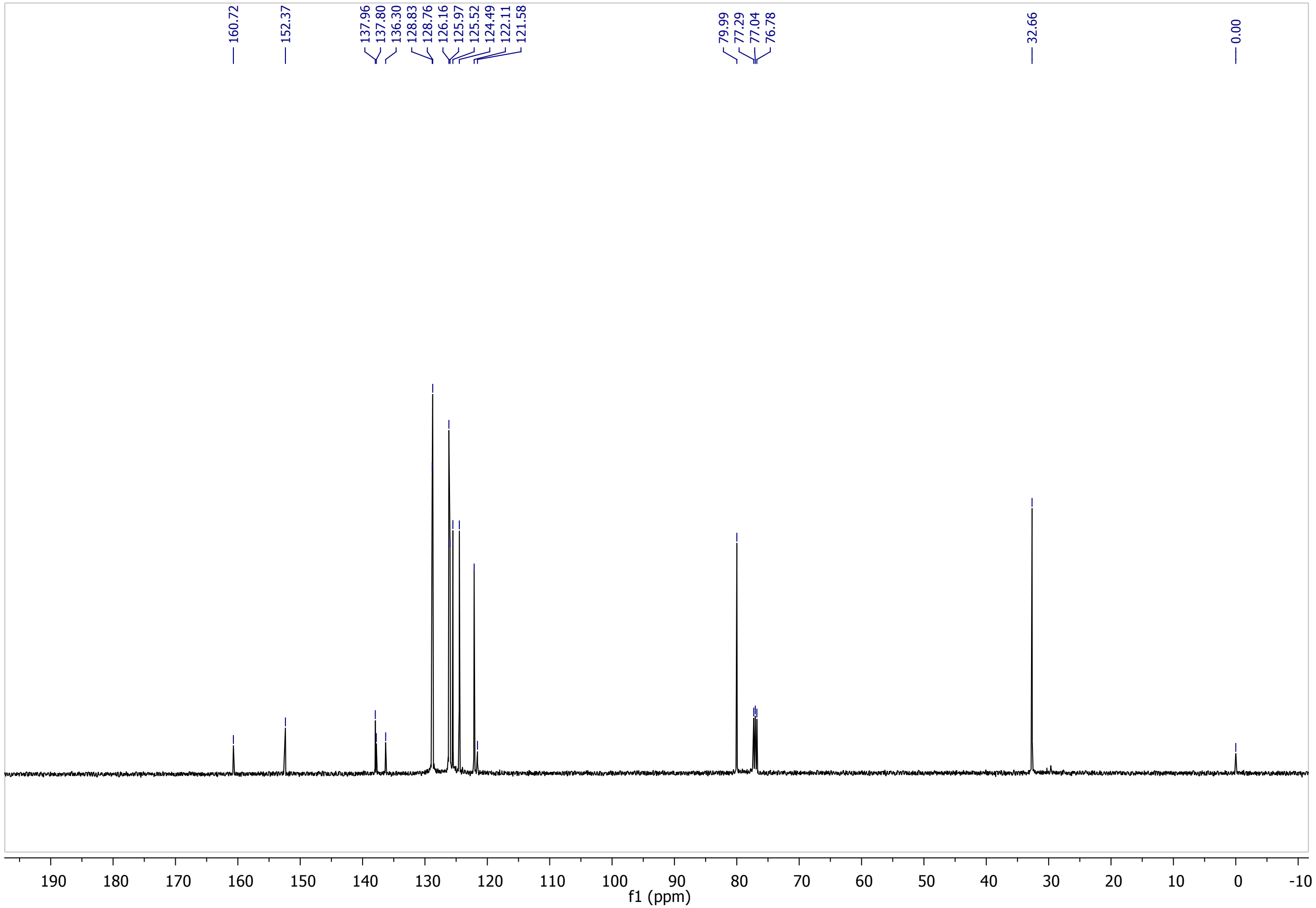

Figure S 28. 3-Phenyl-3,4-dihydro-1*H*-benzo[4,5]thieno[3,2-*c*]pyran-1-one (**A$_4$**)

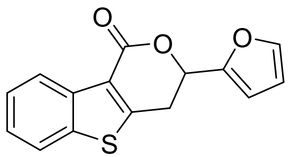

^{1}H NMR ($CDCl_3$, 500 MHz)

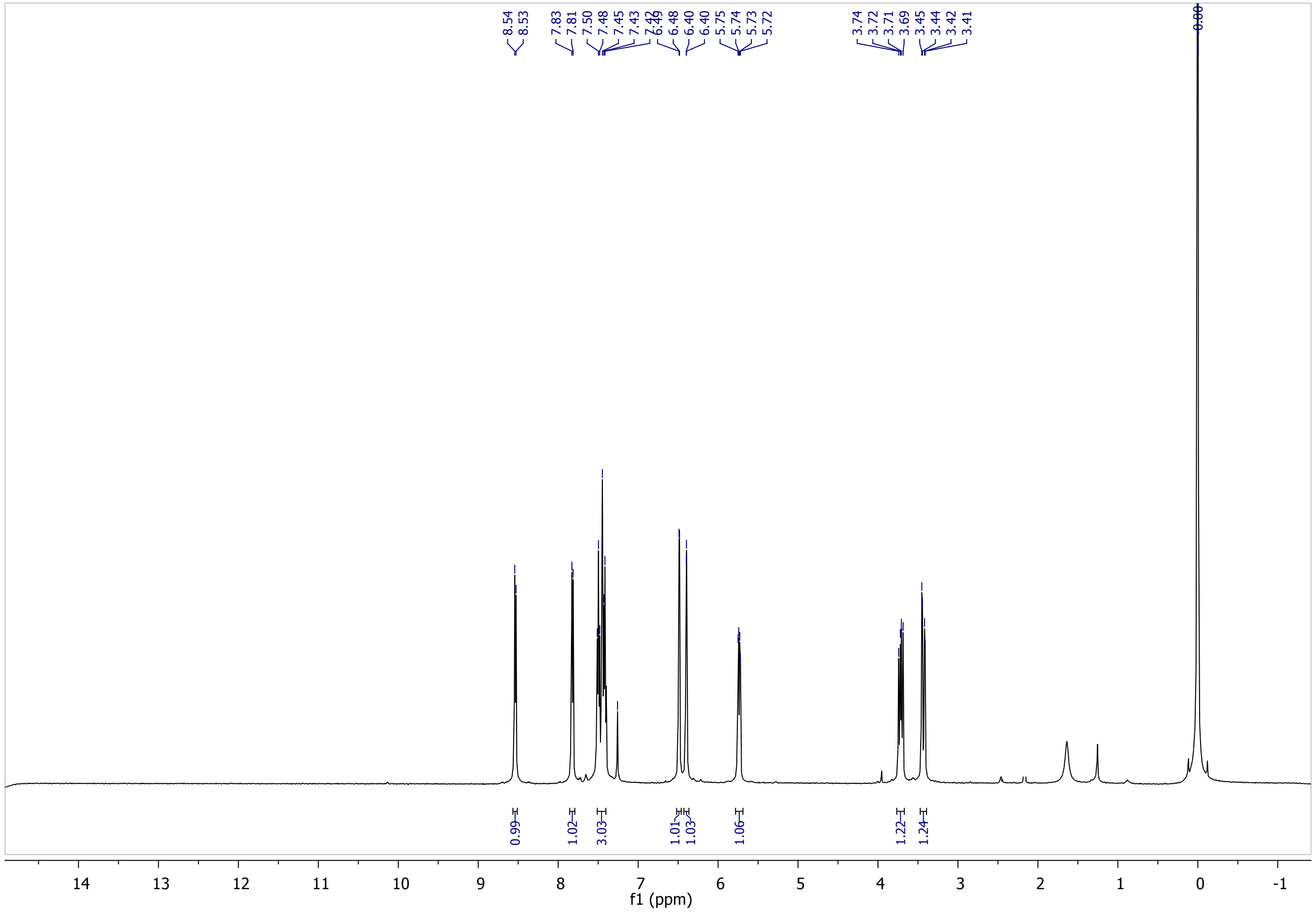

Figure S 29. 3-(Furan-2-yl)-3,4-dihydro-1*H*-benzo[4,5]thieno[3,2-*c*]pyran-1-one (**A$_5$**)

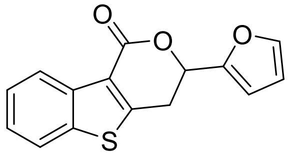

^{13}C NMR ($CDCl_3$, 125 MHz)

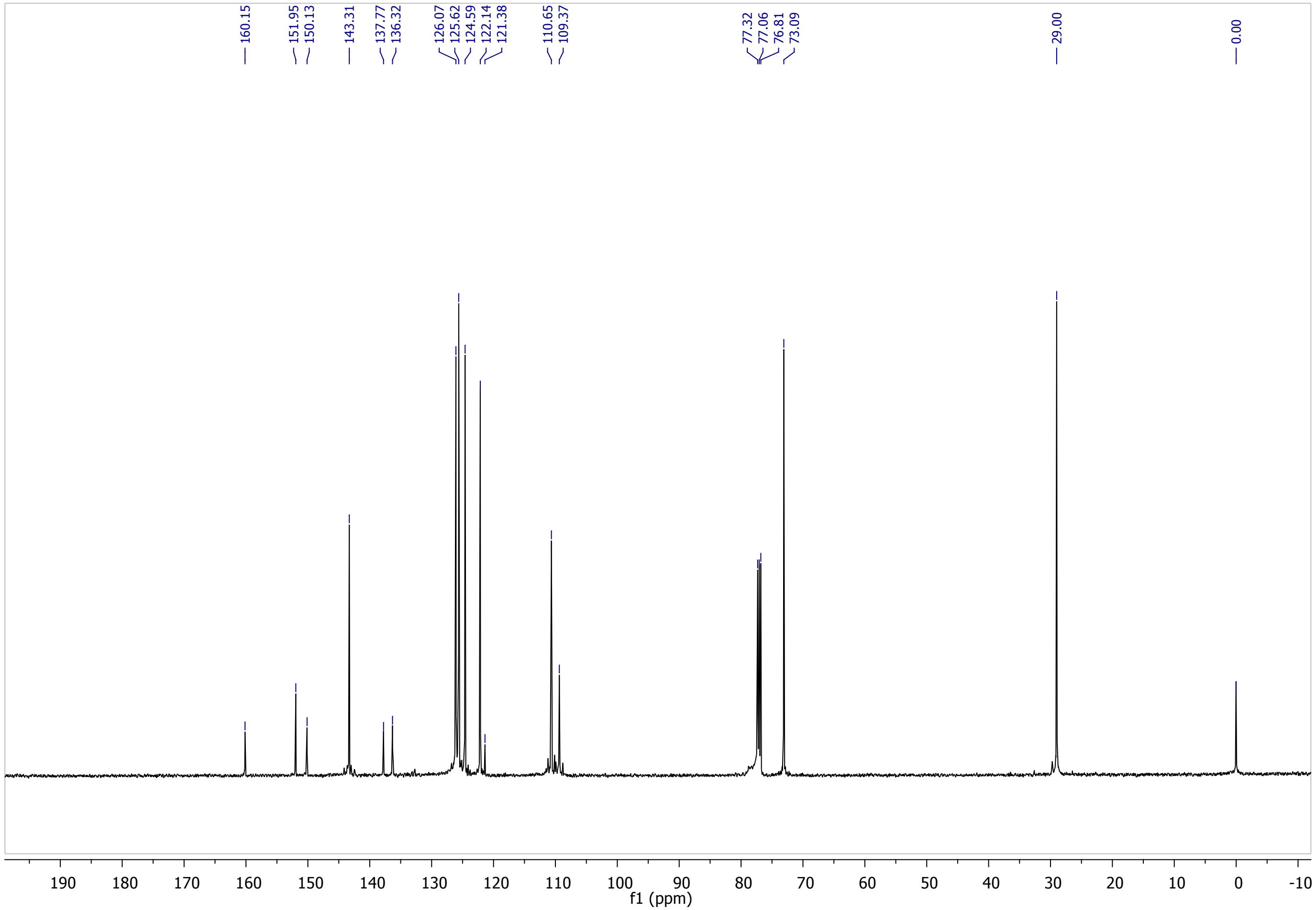

Figure S 30. 3-(Furan-2-yl)-3,4-dihydro-1*H*-benzo[4,5]thieno[3,2-*c*]pyran-1-one (**A$_5$**)
